# Tracking health commodity inventory and notifying stock levels via mobile devices: a mixed methods systematic review

**DOI:** 10.1002/14651858.CD012907.pub2

**Published:** 2020-10-28

**Authors:** Smisha Agarwal, Claire Glenton, Nicholas Henschke, Tigest Tamrat, Hanna Bergman, Marita S Fønhus, Garrett L Mehl, Simon Lewin

**Affiliations:** Department of International HealthJohns Hopkins Bloomberg School of Public HealthBaltimore, MDMaryland (MD)USA; Norwegian Institute of Public HealthOsloNorway; Cochrane ResponseCochraneLondonUK; Department of Sexual and Reproductive HealthWorld Health OrganizationGenevaSwitzerland; Health Systems Research UnitSouth African Medical Research CouncilCape TownSouth Africa

**Keywords:** Bias, Cell Phone, Computers, Handheld, Controlled Before-After Studies, Controlled Before-After Studies/statistics & numerical data, Drugs, Essential, Drugs, Essential/supply & distribution, Equipment and Supplies, Hospital, Equipment and Supplies, Hospital/supply & distribution, Health Personnel, Health Personnel/statistics & numerical data, Interrupted Time Series Analysis, Inventories, Hospital, Inventories, Hospital/methods, Materials Management, Hospital, Materials Management, Hospital/methods, Non-Randomized Controlled Trials as Topic, Non-Randomized Controlled Trials as Topic/statistics & numerical data, Randomized Controlled Trials as Topic, Randomized Controlled Trials as Topic/statistics & numerical data

## Abstract

**Background:**

Health systems need timely and reliable access to essential medicines and health commodities, but problems with access are common in many settings. Mobile technologies offer potential low‐cost solutions to the challenge of drug distribution and commodity availability in primary healthcare settings. However, the evidence on the use of mobile devices to address commodity shortages is sparse, and offers no clear way forward.

**Objectives:**

*Primary objective*

To assess the effects of strategies for notifying stock levels and digital tracking of healthcare‐related commodities and inventory via mobile devices across the primary healthcare system

*Secondary objectives*

To describe what mobile device strategies are currently being used to improve reporting and digital tracking of health commodities

To identify factors influencing the implementation of mobile device interventions targeted at reducing stockouts of health commodities

**Search methods:**

We searched CENTRAL, MEDLINE Ovid, Embase Ovid, Global Index Medicus WHO, POPLINE K4Health, and two trials registries in August 2019. We also searched Epistemonikos for related systematic reviews and potentially eligible primary studies. We conducted a grey literature search using mHealthevidence.org, and issued a call for papers through popular digital health communities of practice. Finally, we conducted citation searches of included studies. We searched for studies published after 2000, in any language.

**Selection criteria:**

For the primary objective, we included individual and cluster‐randomised trials, controlled before‐after studies, and interrupted time series studies. For the secondary objectives, we included any study design, which could be quantitative, qualitative, or descriptive, that aimed to describe current strategies for commodity tracking or stock notification via mobile devices; or aimed to explore factors that influenced the implementation of these strategies, including studies of acceptability or feasibility.

We included studies of all cadres of healthcare providers, including lay health workers, and others involved in the distribution of health commodities (administrative staff, managerial and supervisory staff, dispensary staff); and all other individuals involved in stock notification, who may be based in a facility or a community setting, and involved with the delivery of primary healthcare services.

We included interventions aimed at improving the availability of health commodities using mobile devices in primary healthcare settings. For the primary objective, we included studies that compared health commodity tracking or stock notification via mobile devices with standard practice. For the secondary objectives, we included studies of health commodity tracking and stock notification via mobile device, if we could extract data relevant to our secondary objectives.

**Data collection and analysis:**

For the primary objective, two authors independently screened all records, extracted data from the included studies, and assessed the risk of bias. For the analyses of the primary objectives, we reported means and proportions where appropriate. We used the GRADE approach to assess the certainty of the evidence, and prepared a 'Summary of findings' table. For the secondary objective, two authors independently screened all records, extracted data from the included studies, and applied a thematic synthesis approach to synthesise the data. We assessed methodological limitation using the Ways of Evaluating Important and Relevant Data (WEIRD) tool. We used the GRADE‐CERQual approach to assess our confidence in the evidence, and prepared a 'Summary of qualitative findings' table.

**Main results:**

*Primary objective*

For the primary objective, we included one controlled before‐after study conducted in Malawi.

We are uncertain of the effect of cStock plus enhanced management, or cStock plus effective product transport on the availability of commodities, quality and timeliness of stock management, and satisfaction and acceptability, because we assessed the evidence as very low‐certainty. The study did not report on resource use or unintended consequences.

*Secondary objective*

For the secondary objectives, we included 16 studies, using a range of study designs, which described a total of eleven interventions. All studies were conducted in African (Tanzania, Kenya, Malawi, Ghana, Ethiopia, Cameroon, Zambia, Liberia, Uganda, South Africa, and Rwanda) and Asian (Pakistan and India) countries.

Most of the interventions aimed to make data about stock levels and potential stockouts visible to managers, who could then take corrective action to address them.

We identified several factors that may influence the implementation of stock notification and tracking via mobile device.

These include challenges tied to infrastructural issues, such as poor access to electricity or internet, and broader health systems issues, such as drug shortages at the national level which cannot be mitigated by interventions at the primary healthcare level (low confidence). Several factors were identified as important, including strong partnerships with local authorities, telecommunication companies, technical system providers, and non‐governmental organizations (very low confidence); availability of stock‐level data at all levels of the health system (low confidence); the role of supportive supervision and responsive management (moderate confidence); familiarity and training of health workers in the use of the digital devices (moderate confidence); availability of technical programming expertise for the initial development and ongoing maintenance of the digital systems (low confidence); incentives, such as phone credit for personal use, to support regular use of the system (low confidence); easy‐to‐use systems built with user participation (moderate confidence); use of basic or personal mobile phones to support easier adoption (low confidence); consideration for software features, such as two‐way communication (low confidence); and data availability in an easy‐to‐use format, such as an interactive dashboard (moderate confidence).

**Authors' conclusions:**

We need more, well‐designed, controlled studies comparing stock notification and commodity management via mobile devices with paper‐based commodity management systems. Further studies are needed to understand the factors that may influence the implementation of such interventions, and how implementation considerations differ by variations in the intervention.

## Plain language summary

**Using mobile phones to keep track of medicines and notify superiors when more are needed**

**What is the aim of this review?**

In this Cochrane Review, we aimed to assess if health workers would have better access to medicines and other supplies, if they used mobile phones or other mobile devices to keep track of the amount of supplies they had available, and to notify superiors when they needed more supplies. We also described how health workers are currently using these mobile systems, and what factors influence their use.

**Key messages**

We do not know if using mobile phones to keep track of medical supplies gives primary healthcare workers better access to these supplies. This is because the certainty of the available evidence was very low. However, the evidence does point to several factors that could influence the implementation of these systems. These include the sharing of data across all levels of the health system; reliable access to electricity and the internet; easy‐to‐use and functional phones and systems; good technical support; and sufficient training and supervision. Well‐functioning digital systems will only succeed if medical supplies are actually available.

**What was studied in the review?**

Healthcare workers need medicines, vaccines, syringes, and other supplies to do their jobs properly. But many healthcare settings, particularly in poor countries, often lack supplies because governments cannot afford to buy them, or because they do not have good systems for distributing them to the right places at the right time.

To address some of these problems, managers and health workers can use mobile phones to keep track of supplies, decide how much more to order and when, and let people at higher levels of the system know when supplies are running low.

The main aim of our review was to find out if health workers who use these mobile systems had better access to supplies than health workers using other systems, or no systems at all. We also looked at how these mobile systems are being used in primary healthcare settings, and the factors that influences their use.

**What are the main results of the review?**

We found one study from Malawi that measured the effect of mobile phone systems on access to medicines and supplies in health facilities. However, we do not know whether these systems improve access or not, because the certainty of this evidence was very low.

We found 16 studies conducted in African and Asian countries, in which study authors identified several factors that could help to set up systems successfully.

‐ Study authors suggested that allowing healthcare officials at all levels of the healthcare system to see the data could help them to respond on time and avoid supply shortages (low confidence).

‐ Study authors highlighted how poor access to electricity and internet could make it difficult for health workers to charge phones and send data (moderate confidence).

‐ Study authors pointed to the importance of user‐friendly systems, built with user participation (moderate confidence); access to technical experts to develop and maintain the system (low confidence); proper support and supervision for health workers (moderate confidence); and training of health workers in how to use the mobile system itself (moderate confidence).

‐ One author suggested that adding phone credits to health workers’ mobile phones for their personal use, could motivate them, and improve their use of the system (low confidence). Study authors also suggested several mobile phone functions that could be helpful to health workers using these systems. These included phones that could take pictures and map geographic locations, systems that allowed toll‐free text messaging, two‐way communication, and easy sharing of information (low confidence). But study authors also suggested that health workers might find it easier to use basic phones and maintain their own personal phones (low confidence).

‐ Study authors emphasised that digital notification systems will only give health workers better access to supplies if the supplies are actually available at the district or national level (low confidence).

**How up‐to‐date is this review?**

We searched for studies that had been published up to August 2019.

## Summary of findings

**Summary of findings 1:** Primary objective: mobile stock notification with enhanced management compared to standard care

**Mobile stock notification (cStock) with enhanced management (EM) compared to standard care in primary healthcare settings**						
**Patient or population**: healthcare workers and other health professionals involved in commodity or stock management **Setting**: primary healthcare setting in Malawi **Intervention**: mobile stock notification with enhanced management (cStock + EM), which involved quality improvement teams tasked with using the data supplied by the stock notification system **Comparison**: standard care, which involved routine stock management with mobile stock notification, or any other digital intervention						
**Outcomes**	Illustrative comparative risks* (95% CI)		**Relative effect** **(95% CI)**	**No of participants** **(studies)**	**Certainty of the evidence** **(GRADE)**	**Results in words**
	**Assumed risk with standard care**	**Corresponding risk with cStock + EM**				
**Availability of commodities**						
**Proportion of healthcare workers who reported a stockout of drugs in the last 30 days** *(stockout of cotrimoxazole to treat bacterial infections)*	167 per 1000 healthcare workers	160 per 1000 healthcare workers (82 to 317)	RR 0.96 (0.49 to 1.90)	171 (1 CBA)^a^	⊕⊝⊝⊝ Very low^b,c^	We are uncertain of the effect of this approach on stockout of cotrimoxazole because it is supported by very low‐certainty evidence.
**Proportion of healthcare workers who reported a stockout of drugs in the last 30 days** *(stockout of artemether‐lumefantrine 2 X 6 to treat malaria caused by Plasmodium facilparum)*	189 per 1000 healthcare workers	136 per 1000 healthcare workers (68 to 272)	RR 0.72 (0.36 to 1.44)	171 (1 CBA)^a^	⊕⊝⊝⊝ Very low^b,c^	We are uncertain of the effect of this approach on stockout of artemether‐lumefantrine because it is supported by very low‐certainty evidence.
**Proportion of healthcare workers who reported a stockout of drugs in the last 30 days** *(stockout of oral rehydration salts (ORS) to treat dehydration)*	256 per 1000 healthcare workers	258 per 1000 healthcare workers (156 to 432)	RR 1.01 (0.61 to 1.69)	171 (1 CBA)^a^	⊕⊝⊝⊝ Very low^b,c^	We are uncertain of the effect of this approach on stockout of oral rehydration salts because it is supported by very low‐certainty evidence.
**Proportion of healthcare workers who reported a stockout of drugs in the last 30 days** *(stockout of zinc 20 mg to treat diarrhoea)*	211 per 1000 healthcare workers	209 per 1000 healthcare workers (118 to 376)	RR 0.99 (0.56 to 1.78)	171 (1 CBA)^a^	⊕⊝⊝⊝ Very low^b,c^	We are uncertain of the effect of this approach on stockout of zinc because it is supported by very low‐certainty evidence.
**Quality of stock management**						
**Quality of data about stock management** *(assessed by the extent to which HSAs (intervention group participants) sent messages about the stocks on hand for all the products they managed)*	In the intervention group, an average of 85% (N = 393) of the health surveillance assistants (HSA) who managed relevant medicines reported completely on stock levels. This outcome was not assessed in the comparison group.			393^d^ (1 CBA)^a^	⊕⊝⊝⊝ Very low^b,c^	We are uncertain of the effect of this approach on quality of data about stock management because it is supported by very low‐certainty evidence.
**Timeliness of stock management**						
**Time between stock‐level reporting and appropriate action** *(measured over an 18‐month period (January 2012 to June 2013)*	In the intervention group, health facilities took an average of 12.8 days to fulfil an order requested by the health surveillance assistants (lead time). This outcome was not assessed in the comparison group.			393^d^ (1 CBA)^a^	⊕⊝⊝⊝ Very low^b,c^	We are uncertain of the effect of this approach on the timeliness of stock management because it is supported by very low‐certainty evidence.
**Satisfaction and acceptability**						
**Provider acceptability and satisfaction** *(proportion of participants who reported using the digital intervention)*	In the intervention group, the proportion of participants who reported using the digital intervention (cStock) as the primary means for ordering health products was 97% (N = 81). This outcome was not assessed in the comparison group.			81^d^ (1 CBA)^a^	⊕⊝⊝⊝ Very low^e^	We are uncertain of the effect of this approach on provider satisfaction with stock management because it is supported by very low‐certainty evidence.
**Resource use**						
No studies were identified that reported on this outcome						
**Unintended consequences**						
No studies were identified that reported on this outcome						
*The basis for the assumed risk (e.g. the median control group risk across studies) is provided in footnotes. The corresponding risk (and its 95% confidence interval) is based on the assumed risk in the comparison group and the relative effect of the intervention (and its 95% CI). **95% CI**: 95% confidence interval; **RR:** risk ratio; **CBA**: controlled before‐after trial						
GRADE Working Group grades of evidence **High certainty:** Further research is very unlikely to change our confidence in the estimate of effect. **Moderate certainty:** Further research is likely to have an important impact on our confidence in the estimate of effect and may change the estimate. **Low certainty:** Further research is very likely to have an important impact on our confidence in the estimate of effect and is likely to change the estimate. **Very low certainty:** We are very uncertain about the estimate.						

^a^Shieshia 2014. Published and unpublished data. Study conducted in primary healthcare setting ^b^Downgraded two levels for very serious concerns regarding risk of bias: unclear random sequence generation, allocation concealment, and blinding of participants not feasible given the intervention, unclear blinding of outcomes and incomplete outcome reporting ^c^Downgraded one level for imprecision: small sample size ^d^For this outcome, the number of study participants was based on a different study sample to the one used for the other outcomes. These data come from ongoing data (backend data in a digital system), and comprise of all the health workers who ever reported on stock levels ^e^Non‐comparable results, thus downgraded to very low

**Summary of findings 2:** Primary objective: mobile stock notification with effective product transport compared to standard care

**Mobile stock notification (cStock) with effective product transport (EPT) compared to standard care in primary healthcare settings**						
**Patient or population**: healthcare workers and other health professionals involved in commodity and stock management **Setting**: primary healthcare settings in Malawi **Intervention**: mobile stock notification with effective product transport (cStock + EPT), which involved providing health surveillance assistants (HSA) with training and tools for bicycle maintenance **Comparison**: standard care, involved routine stock management with mobile stock notification or any other digital intervention						
**Outcomes**	Illustrative comparative risks* (95% CI)		**Relative effect** **(95% CI)**	**No of Participants** **(studies)**	**Certainty of the evidence** **(GRADE)**	**Results in words**
	**Assumed risk with standard care**	**Corresponding risk with cStock + EPT**				
**Availability of commodities**						
**Proportion of healthcare workers who reported a stockout of drugs in the last 30 days** *(stockout of cotrimoxazole to treat bacterial infections)*	167 per 1000 healthcare workers	218 per 1000 healthcare workers (117 to 407)	RR 1.31 (0.70 to 2.44)	168 (1 CBA)^a^	⊕⊝⊝⊝ Very low^b,c^	We are uncertain of the effect of this approach on stockout of cotrimoxazole because it is supported by very low‐certainty evidence.
**Proportion of healthcare workers who reported a stockout of drugs in the last 30 days** *(stockout of artemether‐lumefantrine 2 X 6 to treat malaria caused by Plasmodium facilparum)*	189 per 1000 healthcare workers	270 per 1000 healthcare workers (153 to 472)	RR 1.43 (0.81 to 2.50)	168 (1 CBA)^a^	⊕⊝⊝⊝ Very low^b,c^	We are uncertain of the effect of this approach on stockout of artemether‐lumefantrine because it is supported by very low‐certainty evidence.
**Proportion of healthcare workers who reported a stockout of drugs in the last 30 days** *(stockout of oral rehydration salts (ORS) to treat dehydration)*	211 per 1000 healthcare workers	129 per 1000 healthcare workers (63 to 260)	RR 0.61 (0.30 to 1.23)	168 (1 CBA)^a^	⊕⊝⊝⊝ Very low^b,c^	We are uncertain of the effect of this approach on stockout of oral rehydration salts because it is supported by very low‐certainty evidence
**Proportion of healthcare workers who reported a stockout of drugs in the last 30 days** *(stockout of zinc 20 mg to treat diarrhoea)*	256 per 1000 healthcare workers	281 per 1000 healthcare workers (171 to 465)	RR 1.10 (0.67 to 1.82)	168 (1 CBA)^a^	⊕⊝⊝⊝ Very low^b,c^	We are uncertain of the effect of this approach on stockout of zinc because it is supported by very low‐certainty evidence
**Quality of stock management**						
**Quality of data about stock management** *(assessed by the extent to which HSAs (intervention group participants) send messages about the stocks on hand for all the products they managed)*	In the intervention group, an average of 65% (N = 253) of the health surveillance assistants (HSAs) who managed relevant medicines reported on stock levels. This outcome was not assessed in the comparison group.			253^d^ (1 CBA)^a^	⊕⊝⊝⊝ Very low^b,c^	We are uncertain of the effect of this approach on quality of data about stock management because it is supported by very low‐certainty evidence
**Timeliness of stock management**						
**Time between stock‐level reporting and appropriate action** *(Measured over an 18‐month period: January 2012 to June 2013)*	In the intervention group, health facilities took an average of 26 days to fulfil an order requested by the health surveillance assistants (lead time). This outcome was not assessed in the comparison group.			253^d^ (1 CBA)^a^	⊕⊝⊝⊝ Very low^b,c^	We are uncertain of the effect of this approach on the timeliness of stock management because it is supported by very low‐certainty evidence
**Satisfaction and acceptability**						
**Provider acceptability and satisfaction** *(Proportion of participants who reported using the digital intervention)*	In the intervention group, the proportion of participants who reported using the digital intervention (cStock) as the primary means for ordering health products was 91% (N = 78). This outcome was not assessed in the comparison group.			78^d^ (1 CBA)^a^	⊕⊝⊝⊝ Very low^b,c^	We are uncertain of the effect of this approach on provider satisfaction with stock management because it is supported by very low‐certainty evidence
**Resource use**						
No studies were identified that reported on this outcome						
**Unintended consequences**						
No studies were identified that reported on this outcome						
The basis for the assumed risk (e.g. the median control group risk across studies) is provided in footnotes. The corresponding risk (and its 95% confidence interval) is based on the assumed risk in the comparison group and the relative effect of the intervention (and its 95% CI). **95% CI:** 95% confidence interval; **RR:** risk ratio; **CBA**: controlled before‐after trial						
GRADE Working Group grades of evidence **High certainty.** Further research is very unlikely to change our confidence in the estimate of effect. **Moderate certainty.** Further research is likely to have an important impact on our confidence in the estimate of effect and may change the estimate. **Low certainty.** Further research is very likely to have an important impact on our confidence in the estimate of effect and is likely to change the estimate. **Very low certainty.** We are very uncertain about the estimate.						

^a^Shieshia 2014 published and unpublished data. Study conducted in primary healthcare setting. ^b^Downgraded two levels for very serious risk of bias concerns: unclear random sequence generation, allocation concealment, and blinding of participants not feasible given the intervention, unclear blinding of outcomes and incomplete outcome reporting ^c^Downgraded one step for imprecision: small sample size ^d^For this outcome, the number of study participants is based on a different study sample to the one used for the other outcomes. These data come from ongoing data (backend data in a digital system), and comprise all the health workers who ever reported on stock levels.

**Summary of findings 3:** Secondary objective: summary of findings

**Summary of qualitative findings for the secondary objectives^a^**				
	**Summary of review finding**	**Studies contributing to the review finding**	**Overall GRADE‐CERQual assessment of confidence in the evidence**	**Explanation of GRADE‐CERQual assessment^b^**
1	Infrastructural issues, such as challenges in charging phones, uploading and transmitting data, and loss of data due to poor access to electricity and poor or non‐existent internet connectivity were identified as key barriers to implementation.	Negandhi 2016Asiimwe 2011; Atnafu 2017; Biemba 2017; Negandhi 2016; Shieshia 2014; Stanton 2016; USAID 2010Atnafu 2017	Moderate confidence	Reduced due to serious concerns regarding methodological limitations
2	Concern was expressed that digital stock notification systems used at the facility level could not mitigate several, broader health system problems, including an underlying lack of stock at the national or district level, and a mismatch between national ordering routines and local needs.	Chandani 2017; Githinji 2013; Hara 2017; Mikkelsen‐Lopez 2014	Low confidence	Reduced due to serious concerns regarding methodological limitations, and minor concerns regarding adequacy
3	Programmes could benefit from strong partnerships, such as with local authorities; with local telecommunications companies; with technical system providers; and with non‐governmental organizations (NGOs).	WHO 2013	Very low confidence	Reduced due to serious concerns regarding methodological limitations, and serious concerns regarding adequacy
4	The availability and use of data on stock levels at all levels of the health system allowed health care officials to respond to anticipated shortages.	Asiimwe 2011; Barron 2016; Biemba 2017; Shieshia 2014; Stanton 2016	Low confidence	Reduced due to serious concerns regarding methodological limitations, and concerns regarding coherence
5	Supportive supervision and responsive management played an important role in effective adoption of a digital system.	Asiimwe 2011; Barrington 2010; Chandani 2017; Negandhi 2016; Shieshia 2014Asiimwe 2011	Moderate confidence	Reduced due to serious concerns regarding methodological limitations
6	The extent to which health workers are familiar with smartphones and are given adequate training in using the digital system, influences the adoption of the system.	Asiimwe 2011; Barrington 2010; Githinji 2013; Negandhi 2016; Shieshia 2014; Stanton 2016	Moderate confidence	Reduced due to serious concerns regarding methodological limitations
7	The availability of technical programming expertise for the initial development and ongoing maintenance of the digital system is an important implementation factor.	Asiimwe 2011; Biemba 2017; USAID 2010Biemba 2017	Low confidence	Reduced due to serious concerns regarding methodological limitations, and concerns regarding adequacy
8	Incentives, such as receiving phone talk‐time credit, to improve adoption and use of the digital system are valuable.	Barrington 2010	Low confidence	Reduced due to serious concerns regarding methodological limitations, and concerns regarding adequacy
9	User‐friendly systems, built with user participation with easy‐to‐use interfaces were considered important to implementation.	Namisango 2016; Negandhi 2016; Shieshia 2014Namisango 2016	Moderate confidence	Reduced due to serious concerns regarding methodological limitations
10	The use of basic mobile phones or personal phones by health workers reduced challenges with data coverage and expense, and supported easier adoption of the intervention due to familiarity with the phones.	Barrington 2010; Stanton 2016	Low confidence	Reduced due to serious concerns regarding methodological limitations, and minor concerns regarding adequacy
11	Software features, such as ability to capture images, map geographic features, support two‐way communication, toll‐free text messaging, and interoperability were considered important.	Barrington 2010; Negandhi 2016; Shieshia 2014	Low confidence	Reduced due to serious concerns regarding methodological limitations, and minor concerns regarding adequacy
12	Dashboard design and data visualisation played important roles in effective implementation. Managers should have access to data in an easy‐to‐use format, such as an interactive dashboard.	Negandhi 2016; Shieshia 2014; USAID 2010	Moderate confidence	Reduced due to serious concerns regarding methodological limitations

^a^The study authors referred to several factors that may influence the implementation, uptake, or efficient use of interventions targeted at improving stock management ^b^The GRADE‐CERQual evidence profile for each finding is available in Table 4

**1:** GRADE‐CERQual evidence profile

**Summary of review finding**	**Studies contributing to the review finding**	**Methodological limitations**	**Coherence**	**Adequacy**	**Relevance**	**GRADE‐CERQual assessment of confidence in the evidence**	**Explanation of GRADE‐CERQual assessment**
1. Infrastructural issues, such as challenges in charging phones, uploading and transmitting data, and loss of data due to poor access to electricity and poor or non‐existent internet connectivity were identified as key barriers to implementation.	Asiimwe 2011; Atnafu 2017; Biemba 2017; Negandhi 2016; Shieshia 2014; Stanton 2016; USAID 2016	Serious concerns, because 6 studies had serious methodological limitations (insufficient evidence to support findings), and one study had minor methodologic limitations	No, or very minor concerns about coherence	No, or very minor concerns about adequacy	No, or very minor concerns about relevance	Moderate confidence	Reduced due to methodological limitations, as all source material did not include empirical data.
2. Concern was expressed that digital stock notification systems used at the facility level could not mitigate several, broader health system problems, including an underlying lack of stock at the national or district level, and a mismatch between national ordering routines and local needs.	Chandani 2017; Githinji 2013; Hara 2017; Mikkelsen‐Lopez 2014	Serious concerns, because 3 studies had serious methodological limitations (insufficient evidence to support findings), and one study had minor methodologic limitations	No, or very minor concerns about coherence	Minor concerns about adequacy, as 3 studies refer to the same intervention administered in different settings	No, or very minor concerns about relevance	Low confidence	Reduced due to methodological limitations, and concerns about adequacy, as conclusions are based on few studies.
3. Programmes could benefit from strong partnerships, such as with local authorities; with local telecommunications companies; with technical system providers; and with non‐governmental organizations (NGOs).	WHO 2013	Serious concerns, because one study had serious methodological limitations (insufficient evidence to support findings, and unclear description of the intervention)	No, or very minor concerns about coherence	Concerns about adequacy, as only one study contributed to the finding	No, or very minor concerns about relevance	Very low confidence	Reduced due to methodological limitation, as source material did not include empirical data, and concerns about adequacy, as conclusions are based on one study with thin data
4. The availability and use of data on stock levels at all levels of the health system allowed health care officials to respond to anticipated shortages.	Asiimwe 2011; Barron 2016; Biemba 2017; Shieshia 2014; Stanton 2016	Serious concerns, because 4 studies had serious methodological limitations (insufficient evidence to support findings), and one study had minor methodologic limitations	Concerns about coherence due to contradictory findings	No, or very minor concerns about adequacy	No, or very minor concerns about relevance	Low confidence	Reduced due to methodological limitations, and concerns about coherence of the data.
5. Supportive supervision and responsive management played an important role in effective adoption of a digital system.	Asiimwe 2011; Barrington 2010; Chandani 2017; Negandhi 2016; Shieshia 2014	Serious concerns, because 4 studies had serious methodological limitations (insufficient evidence to support findings), and one study had minor methodologic limitations	No, or very minor concerns about coherence	No, or very minor concerns about adequacy	No, or very minor concerns about relevance	Moderate confidence	Reduced due to concerns about methodological limitations
6. The extent to which health workers are familiar with smartphones and are given adequate training in using the digital system, influences the adoption of the system.	Asiimwe 2011; Barrington 2010; Githinji 2013; Negandhi 2016; Shieshia 2014; Stanton 2016	Serious concerns, because 5 studies had serious methodological limitations (insufficient evidence to support findings), and one study had minor methodologic limitations	No, or very minor concerns about coherence	No, or very minor concerns about adequacy	No, or very minor concerns about relevance	Moderate confidence	Reduced due to concerns about methodological limitations.
7. The availability of technical programming expertise for the initial development and ongoing maintenance of the digital system is an important implementation factor.	Asiimwe 2011; Biemba 2017; USAID 2016	Serious concerns, as 3 studies had serious methodological limitations (insufficient evidence to support findings)	No, or very minor concerns about coherence	Concerns about adequacy, as only 3 studies contributed to the finding, and the presented data are sparse.	No, or very minor concerns about relevance	Low confidence	Reduced due to concerns about methodological limitations and adequacy, as conclusions are based on three studies with thin data.
8. Incentives, such as receiving phone talk‐time credit, to improve adoption and use of the digital system are valuable.	Barrington 2010	Serious concerns, because 1 study had serious methodological limitations (insufficient evidence to support findings)	No, or very minor concerns about coherence	Concerns about adequacy, as only 1 study contributed to the finding	No, or very minor concerns about relevance	Low confidence	Due to methodological limitations and concerns about adequacy, as conclusions are based on few studies.
9. User‐friendly systems, built with user participation with easy‐to‐use interfaces were considered important to implementation.	Namisango 2016; Negandhi 2016; Shieshia 2014	Serious concerns, because 2 studies had serious methodological limitations (insufficient evidence to support findings), and one study had minor methodologic limitations	No, or very minor concerns about coherence	No, or very minor concerns about adequacy	No, or very minor concerns about relevance	Moderate confidence	Reduced due to concerns about methodological limitations.
10. The use of basic mobile phones or personal phones by health workers reduced challenges with data coverage and expense, and supported easier adoption of the intervention due to familiarity with the phones.	Barrington 2010; Stanton 2016	Serious concerns, because 2 studies had serious methodological limitations (insufficient evidence to support findings)	No, or very minor concerns about coherence	Concerns about adequacy, as only 2 studies contributed to the finding	No, or very minor concerns about relevance	Low confidence	Reduced due to methodological limitations, and concerns about adequacy, as conclusions are based on few studies.
11. Software features, such as ability to capture images, map geographic features, support two‐way communication, toll‐free text messaging, and interoperability were considered important.	Barrington 2010; Negandhi 2016; Shieshia 2014	Serious concerns, because 2 studies had serious methodological limitations (insufficient evidence to support findings), and one study had minor methodologic limitations	No, or very minor concerns about coherence	Minor concerns about adequacy, due to few studies and the relevant data are sparse.	No, or very minor concerns about relevance	Low confidence	Due to concerns about methodological limitations, and concerns about adequacy, as conclusions are based on few studies.
12. Dashboard design and data visualisation played important roles in effective implementation. Managers should have access to data in an easy‐to‐use format, such as an interactive dashboard.	Negandhi 2016; Shieshia 2014; USAID 2016	Serious concerns, because 2 studies had serious methodological limitations (insufficient evidence to support findings), and one study had minor methodologic limitations	No, or very minor concerns about coherence	No, or very minor concerns about adequacy	No, or very minor concerns about relevance	Moderate confidence	Reduced due to concerns about methodological limitations.

## Background

Access to medicines and other health commodities remains one of the most serious global public health problems and results in critical gaps in delivery of healthcare services.

### Description of the condition

Reliable availability of health commodities is fundamental to diagnosing and treating illnesses in primary healthcare settings. Health commodities include health products, health and medical supplies, and other items that may be needed for the provision of health services, including medicines; vaccines; medical supplies, such as contraceptives, dressings, needles, and syringes; and laboratory and diagnostic consumables (Tran 2015; WHO 2017). The World Health Organization (WHO) Global Strategy for Women’s and Children’s Health highlights the importance of equitable access to life‐saving medicines and other health commodities (WHO 2010). A hallmark of functioning health systems is the availability of essential medicines in adequate amounts, appropriate dosage forms, and assured quality – at a price that is affordable for the local community (Tran 2015; WHO 2016). However, stockouts of critical medical commodities, such as medicines, are widespread, especially in low‐ and middle‐income countries (LMICs).

At least one third of the world’s population does not have regular access to medicines, which makes health care highly inequitable (WHO 2011). A survey of the national AIDS programmes in 12 countries, by the Pan American Health Organization (PAHO), found that between January 2011 and April 2012, over 67% of the countries reported experiencing stockouts of at least one drug, lasting an average of 40 days each (Sued 2011). Another study, in Kenya, reported that over 75% of health facilities had shortages of one component of the combination of drugs used to treat malaria, while one in four reported a lack of all related drugs (Kangwana 2009). This lack of access to critical drugs, caused by a stockout, has profound effects on the ongoing treatment of diseases. A study in Côte d’Ivoire reported that people who experienced interruptions in their HIV treatment, caused by drug shortages, were twice as likely to permanently discontinue treatment or die (Pasquet 2010). Drug stockouts have been linked to increases in morbidity and mortality across several disease states in low‐resource settings, including malaria (Chuma 2010), HIV (Pasquet 2010), and the prevention or treatment of pregnancy complications (Hill 2006).

Lack of access to medicines and other health commodities is often symptomatic of broader systemic problems. For example, access to medicines is determined by rational use of medicines, affordable pricing, sustainable financing, and reliable health and supply systems (WHO 2004; WHO 2015). A reliable medicine supply system should include appropriate procurement and distribution. A good distribution system ensures timely availability of medicines across all levels of the healthcare system and prevention of stockouts (WHO 2017).

### Description of the intervention

The rapid global expansion of mobile technology has provided a potential low‐cost solution to the challenge of drug distribution and stockouts. Plummeting costs of mobile handsets and services have made mobile phone technology accessible to people living in rural and underserved areas. Mobile interventions may address stockouts of medicines and health commodities primarily through two strategies: supply chain management, and assessment and reporting of essential commodities (Mehl 2017 [pers comm]).

Supply chain management involves approaches for monitoring and reporting stock levels, consumption and distribution of medical commodities, as well as approaches to analyse and project usage of medical commodities. This can include the use of communication systems, such as short message service (SMS) and data dashboards, to manage and report on supply levels of medical commodities. Some specific examples where mobile tools may be used to improve supply chain management include tracking inventory of health commodities, notifying stock levels of health commodities, monitoring cold‐chain sensitive commodities, and managing distribution of health commodities.

Digital approaches for assessment and reporting of essential commodities are often used for reporting and tracking the authenticity and quality of medical commodities. This can include using mobile functions, such as barcode readers and short message service (SMS) communication to validate an authentication code on the drug packaging (Frøen 2016), as well as to report on adverse drug effects. Specific examples where mobile devices may be used for assessment and reporting of commodities include reporting on stock levels, reporting counterfeit or substandard drugs, reporting adverse drug interactions, and registering licensed drugs and health commodities.

### How the intervention might work

Mobile devices are being used for supply chain management to improve data visibility, improve decision‐making, and help to address the availability of commodities. There is a substantial amount of variation in how such systems might operate. At the most basic level, interventions may involve citizens reporting counterfeit medicines, using SMS sent to a toll‐free phone number. Such interventions may use a mobile product authentication (MPA) application, or a barcode that allows consumers to text a set of unique numbers to a toll‐free phone number, to verify if a medicine is authentic. In response, consumers may receive a SMS that indicates the legitimacy of the medicine.

Other interventions may involve frontline healthcare workers or healthcare administrators in primary healthcare settings using mobile devices to collect data on stock levels, so that data can be instantly digitised and used to predict and prevent stockouts, and respond to drug shortages. More comprehensive interventions may aim to develop a technology‐based system for reporting of drug stock levels, and change the culture around the use of data (on stock levels), and accountability for responding to projected shortages. For example, cStock is an open‐source internet‐accessible logistics management information system that targets the availability of health commodities at the community level, in Malawi (Shieshia 2014). Health surveillance assistants (HSAs), who typically deliver primary healthcare services in the community, send information about the amount of medicine stocks they have on hand, via a text message to a toll‐free number. The logistics management system automatically collates this data from multiple HSAs, calculates the total quantities of commodities needed, and sends a text message to the HSAs when the medicines are available at the nearest health centre. These data are also available on a internet‐accessible dashboard, with simple, easy‐to‐use reports, showing stock levels, HSA reporting rates, and alerts from central and district level health managers. Making real‐time data available, with regular reports of stock levels, allows managers and other stakeholders to co‐ordinate, plan, and identify solutions to better meet community needs in a timely manner.

### Why it is important to do this review

There is rapid progress in the use of mobile devices to address systemic challenges in the delivery of healthcare services. Despite the exponential growth of mobile device‐based interventions and their potential, there remain several unanswered questions about the effectiveness of such interventions. The reliable availability of essential medicines and health commodities is foundational to a responsive health system, and an area that is of much interest to governments, especially in LMICs. However, the evidence on the use of mobile devices to address drug and commodity shortages is sparse, and offers no clear way forward. We are not aware of any existing systematic reviews that assess the effectiveness of strategies to improve stock notification, through either digital or non‐digital approaches. The WHO recently published guidelines to inform investments in digital health applications for strengthening health systems (WHO 2019). Through a consultative process, assessing the impact of mobile interventions to address stockouts was identified as one of the several areas to be included in the guidelines. This Cochrane Review is one of a suite of reviews that contributed to these guidelines. We aimed to assess the effectiveness of using mobile devices to address stockouts of drugs and essential health commodities, and the acceptability, resource use, and unintended consequences of such interventions.

## Objectives

### Primary

- To assess the effects of strategies for notifying stock levels and digitally tracking healthcare‐related commodities and inventory, via mobile devices, across the primary healthcare system.

### Secondary

- To describe what mobile device strategies are currently being used to improve reporting and digital tracking of health commodities;
- To identify factors influencing the implementation of mobile device interventions targeted at reducing stockouts of health commodities.

## Methods

### Criteria for considering studies for this review

#### Types of studies

##### Primary objective

For the review’s primary objective, we included these study designs:

- Randomised trials;
- Non‐randomised trials;
- Controlled before‐after studies, provided they had at least two intervention sites and two control sites;
- Interrupted time series studies, if there was a clearly defined point in time when the intervention occurred, and at least three data points before and three after the intervention.

We included published studies, conference abstracts, and unpublished data. We included studies, regardless of their publication status, or language of publication.

##### Secondary objectives

For the review's secondary objectives, we included any studies that used descriptive, qualitative, or quantitative methods to describe interventions that were aimed at improving stockouts of health commodities.

#### Types of participants

For the review's primary and secondary objectives, we included studies with these participants:

- All cadres of healthcare providers (i.e. professionals, para‐professionals, and lay health workers), or others involved in the distribution of health commodities, located at any level of the health system (e.g. administrative staff, managerial and supervisory staff in purchasing or distribution, or dispensary staff);
- Other individuals or groups involved in stock notification, monitoring, and tracking commodity inventories. These individuals or groups may be based in a primary healthcare facility or in the community, and must be involved in supporting the delivery of primary healthcare services.
- Clients or recipients of health services

#### Types of interventions

For the review's primary and secondary objectives, we included interventions that were aimed at improving the availability of health commodities, including medicines and other medical supplies, using mobile devices for the delivery of primary healthcare services in healthcare facilities or in the community, if they involved one or both of the following:

- Strategies for tracking health commodity inventory using mobile devices. Tracking health commodity inventory may have involved the use of databases and dashboards to manage the availability of health commodities and project availability of medical supplies. While some aspects of commodity tracking might have involved mobile devices, the data may have been linked to a logistics management information system (LMIS) or supply chain management system, where inventory levels and historic data were maintained on desktops;
- Notification of stock levels conducted via mobile devices. This may have involved the transmission of information on stock levels by health workers within healthcare facilities or by members of the community, to alert higher‐level facilities about potential stock shortages. For example, health workers at facilities or dispensaries may have used text messaging, short message service (SMSa), or unstructured supplementary service data (USSD)‐based systems to notify district or central authorities about stock levels. In some interventions of interest, notification of stock levels using mobile phones may have been a component of a broader strategy for tracking health commodities.

By mobile devices, we meant mobile phones of any kind (but not analogue landline telephones), tablets, personal digital assistants, and smartphones.

By primary healthcare services, we meant a combination of the following:

- The first contact point of healthcare (Awofeso 2004), including care delivered at an individual or community level, or both, by individual healthcare providers or teams of providers, and intended to bring care to where people worked and lived (Muldoon 2006), or the co‐ordination or provision of continuity of care, or both (WHO 2008);
- Any rehabilitative, therapeutic, preventive, or promotional healthcare (Global Health Watch 2011).

The key comparison for this review was tracking commodity inventory and notifying stock levels via mobile devices compared with standard practice (i.e. non‐digital strategies or no intervention).

We excluded:

- Studies that focused on cold chain management only, and did not report on stock levels of the vaccines;
- Studies where commodity tracking and notification of commodities was conducted on stationary computers or laptops only.

Where tracking or notification via mobile device, or both, was delivered as part of a wider package, we included the study if we judged the mobile component to be the major component of the intervention.

#### Types of outcome measures

##### Primary objective

For the review's primary objective, we included studies that assessed the following outcome measures:

- Availability of commodities, measured, for instance, as decreased stockouts, lead time for drug supply, availability at point of care;
- Quality of data about stock management (accuracy of data, completeness of data);
- Timeliness of stock level reporting, and time between receipt and reporting data regarding commodity status and appropriate action;
- Provider acceptability or satisfaction with the intervention, measured with a validated scale if available;
- Resource use (e.g. human resources or time, including additional time spent by providers when managing or transitioning dual paper and digital reporting systems; training, supplies, and equipment);
- Unintended consequences that may result in the intervention having adverse effects (these could include: misreading or misinterpreting the data; transmitting inaccurate data, for instance through software formatting errors; interrupted workflow due to infrastructure constraints for battery recharge and network coverage; decreased motivation or trust in the system by health workers, if stock replenishment is not reliable; loss or misuse of mobile device).

### Search methods for identification of studies

We started the search in 2000. This was based on the increased availability and penetration of mobile devices in LMICs from 2000 onwards (ITU 2015).

#### Electronic searches

An independent Information Specialist (JE) developed the search strategies in consultation with the review authors.

We searched the following databases for primary studies, from 2000 to the date of search:

- Cochrane *C*entral Register of Controlled Trials (CENTRAL; 2019, Issue 8), in the Cochrane Library (searched 7 August 2019);
- MEDLINE Ovid and Epub Ahead of Print, In‐Process & Other Non‐Indexed Citations and Daily (1946 to 7 August 2019);
- Embase Ovid (1974 to 2019 Week 31);
- POPLINE, K4Health (searched 7 August 2019);
- Global Index Medicus, World Health Organization (WHO; searched 7 August 2019).

Appendix 1 lists the search strategies we used to search all the databases. Search strategies were comprised of keywords and controlled vocabulary terms. We did not apply any limits on language.

#### Searching other resources

##### Trial registries

We searched for ongoing trials in the following trial registries, and contacted authors for further information and data, if available:

- WHO International Clinical Trials Registry Platform (ICTRP; www.who.int/ictrp; searched 7 August 2019);
- US National Institutes of Health Ongoing Trials Register ClinicalTrials.gov (www.clinicaltrials.gov; searched 7 August 2019).

We searched Epistemonikos (www.epistemonikos.org; searched 27 January 2020) for relevant systematic reviews and potentially eligible primary studies. Additionally, the WHO issued a call for papers through popular digital health communities of practice, such as the Global Digital Health Network, to identify additional primary studies and grey literature.

##### Grey literature

We searched www.mhealthevidence.org for grey literature. The search portal for mhealthevidence.org was more limited; therefore, we reviewed the titles and abstracts of all contributed literature that was not referenced in MEDLINE Ovid (searched 15 August 2017; the database was discontinued in 2018).

We reviewed reference lists of all included studies and relevant systematic reviews for additional, potentially eligible primary studies. We also conducted a citation search for studies that had cited any included studies (searched 27 January 2020). We contacted authors of included studies and reviews to clarify reported published information, and to seek unpublished results and data.

### Data collection and analysis

#### Selection of studies

A core team of two authors (NH and HB), with assistance where necessary from one additional review author (SA), were responsible for the selection of studies. We downloaded all titles and abstracts retrieved by electronic searching to a reference management database and removed duplicates. Two review authors (NH and HB) independently screened titles and abstracts for inclusion for the primary and secondary objectives. We retrieved the full‐text study reports and publications for titles and abstracts that were assessed as potentially eligible. Two review authors (NH and HB) for the primary objective, and one review author for the secondary objectives (SA), independently screened the full‐text, and identified studies for inclusion, and recorded reasons for exclusion of the ineligible studies. We resolved any disagreement through discussion; if required, we consulted a third review author.

We listed studies that initially appeared to meet the inclusion criteria but that we later excluded in the 'Characteristics of excluded studies' table. We collated multiple reports of the same study, so that each study, rather than each report, was the unit of interest in the review. We also provided any information we obtained about ongoing studies. We recorded the selection process in sufficient detail to complete a PRISMA flow diagram (Liberati 2009).

#### Data extraction and management

We modified the Cochrane Effective Practice and Organisation of Care (EPOC) standard data collection form and adapted it for study characteristics and outcome data (EPOC 2017a). We identified key characteristics of the intervention for abstraction based on the mHealth Evidence Review and Assessment (mERA) guidelines (Agarwal 2016). We piloted the form on one study in the review.

##### Primary objective

Two review authors (NH and HB) independently extracted the following study characteristics from the studies that were included for the primary objective:

- general information: title, reference details, author contact details, publication type, funding source, conflicts of interest of study authors;
- population and setting: country, geographical location (rural, urban, peri‐urban), healthcare setting (e.g. facility‐based, community‐based);
- methods: function of the intervention, study design, unit of allocation, study duration;
- participant characteristics: type of user (role, if in the health system; length of training, if any), description of any other participants in the intervention, withdrawals;
- interventions: intervention purpose, components, infrastructure to support the technology, type of technology (software platform), type of mobile device(s) used (smartphone, tablets with a screen size larger than 7 inches, feature phones that can run java applications, basic phone with SMS and call functions, laptops), mode of delivery, content of the intervention, participant and provider training, interoperability, compliance with national guidelines, data security, comparison, fidelity assessment, duration of intervention;
- outcomes: primary and other outcomes specified and collected, time points reported, adverse events, results of any subgroup analyses.

We noted in the 'Characteristics of included studies' table if outcome data were reported in a way that was not usable.

##### Secondary objective

For the secondary objectives, we extracted all the information listed above, if available, to describe the intervention. To understand factors affecting the implementation of relevant interventions, we had planned to use the Supporting the Use of Research Evidence (SURE) framework (SURE 2011; Glenton 2017); however, we found that the themes identified in the framework did not apply well to the contents of the included studies. We also explored the use of other implementation research frameworks, such as the consolidated framework for implementation research (CFIR; (Damschroder 2015), but found minimal overlap between the themes identified in the data. Therefore, we read and re‐read the included studies to identify new codes to tag the abstracted data.

#### Assessment of risk of bias in included studies

##### Assessment of risk of bias in included study for the primary objective

For the primary objective, two review authors (NH and HB) independently assessed the risk of bias for the included study, using the criteria outlined in the *Cochrane Handbook for Systematic Reviews of Interventions* Section 8.5 (Higgins 2011), and guidance from the Cochrane EPOC group (EPOC 2017b). We assessed risk of bias for the included controlled before‐after study using the following criteria: random sequence generation, allocation concealment, blinding of participants and personnel, blinding of outcome assessment, incomplete outcome data, selective outcome reporting, baseline outcomes measurement, similarity of baseline characteristics, and other bias.

We judged each potential source of bias as either high, low, or unclear, and provided a quote from the study together with a justification for our judgment, in Table 5. We considered blinding separately for different key outcomes where necessary (e.g. for unblinded outcome assessment, risk of bias for all‐cause mortality may be very different than for a participant‐reported pain scale). When considering treatment effects, we took into account the risk of bias for the study that contributed to that outcome.

**2:** Risk of bias in the included study for the primary objective (Shieshia 2014)

**Bias**	**Authors' judgement**	**Support for judgement**
Random sequence generation (selection bias)	Unclear risk	"randomly assigned", but method of sequence generation not reported
Allocation concealment (selection bias)	Unclear risk	Not reported
Blinding of participants and personnel (performance bias)	High risk	Blinding was not possible due to the nature of the intervention
Blinding of outcome assessment (detection bias)	Unclear risk	Not reported
Incomplete outcome data (attrition bias)	Unclear risk	Different sample sizes for different outcomes; more participants were analysed at follow‐up than at baseline; and only a sample of participants were included in the analysis. It was unclear whether the researchers were able to collect data from all respondents.
Selective reporting (reporting bias)	Low risk	All outcomes in the protocol were reported in the published results
Other bias	Low risk	No other bias identified

##### Assessment of methodological limitations of included studies for the secondary objectives

For the secondary objectives, the included studies comprised a multitude of study designs and study aims, including case studies that were primarily descriptive. We were unable to find an accepted tool designed to appraise methodological limitations that could accommodate this variation in study design. Therefore, we piloted a newly developed tool for assessing the methodological limitations of sources, such as programme reports, that do not use typical empirical research designs. Two review authors (SA and CG) independently assessed the methodological limitations of the studies using the Ways of Evaluating Important and Relevant Data (WEIRD) tool (Lewin 2019). The tool, which is currently being piloted in EPOC and other systematic reviews, is available in Appendix 2.

For each item and question in the tool, the review author selected one of the following response options:

- Yes – the item was addressed adequately in the source
- Unclear – it is not clear if the item was addressed adequately in the source
- No – the item was not addressed adequately in the source
- Not applicable – the item is not relevant to the source being assessed

The assessments for each WEIRD tool item for each relevant study are reported in Table 6.

**3:** Methodological limitations of the included studies for the secondary objectives^a^

**Study ID**	**Clearly stated aim, objective or purpose?**	**Clear description of the source of the information (transparency)?**	**Clear description of the programme,intervention, policy or reform?**	**Clear description of the context/s?**	**Is the information accurate?**	**Is the evidence representative?**	**Any limitations of the information and/or methods discussed?**	**Is evidence provided to support any findings or conclusions made?**	**Relevant rights and ethics considerations described (empirical studies only)**	**Interests declared and any potential conflicts of interest noted?**	**Overall assessment**^d^
Asiimwe 2011	Unclear^b^	Unclear	Yes	Yes	Unclear	Unclear	No	No	N/A	Unclear ‐ funding source declared but no conflict of interest declaration	Major limitations^e,f,g,h^
Atnafu 2017	Yes	Yes	Yes	Yes	Yes	Yes	Yes	Yes	Yes	Yes ‐ none to declare	No or few limitations^e^
Barrington 2010	Unclear^b^	Yes	Yes	Yes	Yes	Yes	Yes	No	N/A	Yes ‐ conflicts reported	Major limitations^e,g,h^
Barron 2016	Unclear^b^	Yes	Yes	Yes	Yes	Yes	Yes	No	N/A	No reporting on conflict of interest	Major limitations^e,g,h^
Biemba 2017	Unclear^b^	Yes	Yes	Yes	Yes	Unclear^c^	Yes	No	N/A	Yes ‐ none to declare	Major limitations^e,g^
Chandani 2017	Yes	Yes	Yes	Yes	Yes	Unclear^c^	Yes	Yes	Yes	No reporting on conflict of interest (however, a related study has reported on COI)	Minor limitations^h,i^
Githinji 2013	Unclear^b^	Yes	Yes	Yes	Yes	Yes	Yes	No	N/A	Yes ‐ conflicts reported	Major limitations^e,g,h^
Hara 2017	Unclear^b^	Yes	Yes	Yes	Yes	Yes	Yes	No	N/A	Yes ‐ none to declare	Major limitations^e,g^
Mikkelsen‐Lopez 2014	Unclear^b^	Yes	Yes	Yes	Yes	Yes	Yes	No	N/A	Yes ‐ conflicts reported	Major limitations^e,g,h^
Namisango 2016	Unclear^b^	Yes	Yes	Yes	Yes	Yes	Yes	No	N/A	Yes ‐ none to declare	Major limitations^e,g^
Negandhi 2016	Yes	Yes	Yes	Yes	Yes	Unclear^c^	Yes	No	N/A	Yes ‐ none to declare	Major limitations^e,g^
Shieshia 2014	Unclear^b^	Yes	Yes	Yes	Yes	Yes	Yes	Yes	Yes	Yes ‐ none to declare	Minor limitations^e^
Stanton 2016	Unclear^b^	Yes	Yes	Yes	Yes	Yes	No	No	N/A	Yes ‐ none to declare	Major limitations^e,g^
USAID 2010	No	No	No	No	No	No	No	No	N/A	No reporting on conflict of interest	Major limitations^e,g,h,i,j^
USAID 2016	No	No	No	No	No	No	No	No	N/A	No reporting on conflict of interest	Major limitations^e,g,h,i,j^
WHO 2013	No	No	No	No	No	No	No	No	N/A	No reporting on conflict of interest	Major limitations^e,g,h,i,j^

^a^Details of the WEIRD tool assessment criteria and prompts are available in Appendix 2 ^b^Stated aim does not include assessing implementation factors ^c^Small sample size ^d^*No or few limitations*: when the answer to most questions in the tool is YES *Minor limitations*: when the answer to most questions in the tool is YES or UNCLEAR *Significant / major limitations*: when the answer to one or more questions in the tool is NO Explanation of overall assessments ^e^Concerns related to relevance of study aim to review objectives ^f^Concerns related to source of information reported ^g^Concerns related to evidence to support findings ^h^Concerns related to lack of COI declaration ^i^Concerns related to generalizability ^j^Concerns related to description of source of information, aims, programme and context

Based on the assessments for each WEIRD tool item, we made an overall assessment of the methodological limitations of the source as follows:

- Where the assessments for most items in the tool were 'yes' – no or few limitations
- Where the assessments for most items in the tool were 'yes' or 'unclear' – minor limitations
- Where the assessments for one or more questions in the tool were 'no' – major limitations

For each source, our assessment of whether most of the WEIRD tool items were addressed or not was a judgement. To make these judgements as explicit and transparent as possible, we have provided explanations of our reasoning in Table 6.

We then used the overall assessment for each source as part of the GRADE‐CERQual assessment of how much confidence to place in the findings for each secondary objective.

#### Measures of treatment effect

For the review's primary objective, we report pre‐intervention and post‐intervention means and proportions for the intervention and comparison groups, where possible. We estimated the effect of the intervention using risk ratios for dichotomous data, together with the appropriate associated 95% confidence interval (CI) and mean difference.

#### Unit of analysis issues

For the controlled before‐after studies included in the review, we had planned to report cluster adjusted risk ratios and their 95% CIs. However, the analysis of the one included cluster trial was not adjusted for clustering, and no intracluster correlation coefficient (ICC) was available (Shieshia 2014). Therefore, we presented the results without a measure of variance or precision of effect for outcomes for which there is a unit of analysis error (EPOC 2017c)

#### Dealing with missing data

We contacted investigators in order to verify key study characteristics and obtain missing outcome data where possible (e.g. when a study was identified as an abstract only).

#### Assessment of heterogeneity

We did not undertake a meta‐analysis, as we only included one study for the primary objective.

#### Assessment of reporting biases

We did not explore reporting bias statistically, as we only included one study for the primary objective.

#### Data synthesis

We presented a narrative overview of the findings, together with tabular summaries of extracted data, for the primary objective. We used Mantel‐Haenszel risk ratios to present results from dichotomous data, where sufficient data were available.

As part of the data synthesis, we had planned to explore how we could integrate the findings from our primary objective with those of the secondary objective. However, this was not feasible, as only one study was eligible for the primary objective and we assessed the findings from the primary objective to be of very low certainty.

For the secondary objectives, we had originally planned to use the SURE framework. However, we found that the themes identified in the framework did not apply well to the contents of the included studies. Therefore, we applied a thematic analysis approach. We read and re‐read the included studies, coded the data, and generated themes. We then identified common themes across all included studies, and consolidated themes where they had overlapping data, and divided themes further if the data captured disparate ideas. Thematic synthesis is a standard approach that has been used across several qualitative evidence summaries. We only reported themes emerging from the data; we did not apply any other organizing frameworks.

Once the review findings were completed, one author went through each finding, identified factors that may influence the implementation of the intervention, and developed prompts for future implementers. These prompts were reviewed by at least one other review author. These prompts are not intended to be recommendations, but instead, are phrased as questions to help implementers consider the implications of the review findings in their context. The questions are presented in the 'Implications for practice' section.

#### Subgroup analysis and investigation of heterogeneity

Sub‐group analysis was not possible as we only included one study for the primary objective, and it did not have data relevant to any planned sub‐group analyses.

#### Sensitivity analysis

We did not identify a sufficient number of studies to perform sensitivity analyses.

#### Summary of findings and assessment of the certainty of the evidence

We created 'Summary of findings' tables for the main intervention comparison(s) and included the most important outcomes in order to draw conclusions about the certainty of the evidence within the text of the review:

- Availability of commodities (e.g. proportion of health workers or facilities reporting drug stockouts, time between stockout and availability of commodities);
- Quality of data about stock management (e.g. accuracy of data, completeness of data);
- Timeliness of stock‐level reporting;
- Provider acceptability or satisfaction with the intervention.
- Resource use
- Unintended consequences

For the primary objective, two review authors independently assessed the certainty of the evidence (high, moderate, low, or very low) using the five GRADE considerations (risk of bias, consistency of effect, imprecision, indirectness, and publication bias) (Guyatt 2008). We used methods and recommendations described in Section 8.5 and Chapter 12 of the *Cochrane Handbook for Systematic Reviews of interventions* (Higgins 2011), and the Cochrane EPOC worksheets (EPOC 2017d), and used GRADEpro software (GRADEpro GDT). We provided justification for decisions to downgrade or upgrade the ratings using footnotes in the table. We used plain language statements to report these findings in the review (EPOC 2017e).

For the secondary objectives, two authors (SA, CG) used the GRADE‐CERQual approach to assess our confidence in each finding (Lewin 2018). GRADE‐CERQual assesses confidence in the evidence, based on the following four key components: methodological limitations of included studies; coherence of the review finding; adequacy of the data contributing to a review finding; and relevance of the included studies to the review question. After assessing each of the four components, we made a judgement about the overall confidence in the evidence supporting the review finding. We judged confidence as high, moderate, low, or very low. The final assessment was based on consensus among the review authors. The GRADE‐CERQual evidence profile tables supporting the assessment of confidence in each finding can be found in Table 4.

## Results

### Description of studies

#### Results of the search

We conducted a systematic literature search to August 2019. We identified a total of 4886 references after removing duplicates. We excluded 4778 references for the primary and secondary objectives, following a review of the titles and abstracts. We retrieved the full texts of 92 articles for the primary and secondary objectives for detailed eligibility assessment.

We included one study that fulfilled our inclusion criteria for the review’s primary objective (Shieshia 2014, published and unpublished data).

We included 16 papers that fulfilled our inclusion criteria for the review’s secondary objectives, including the one study that was also included for the primary objective.

We excluded 76 articles for reasons described in Figure 1. We did not identify any ongoing studies.

**1:**
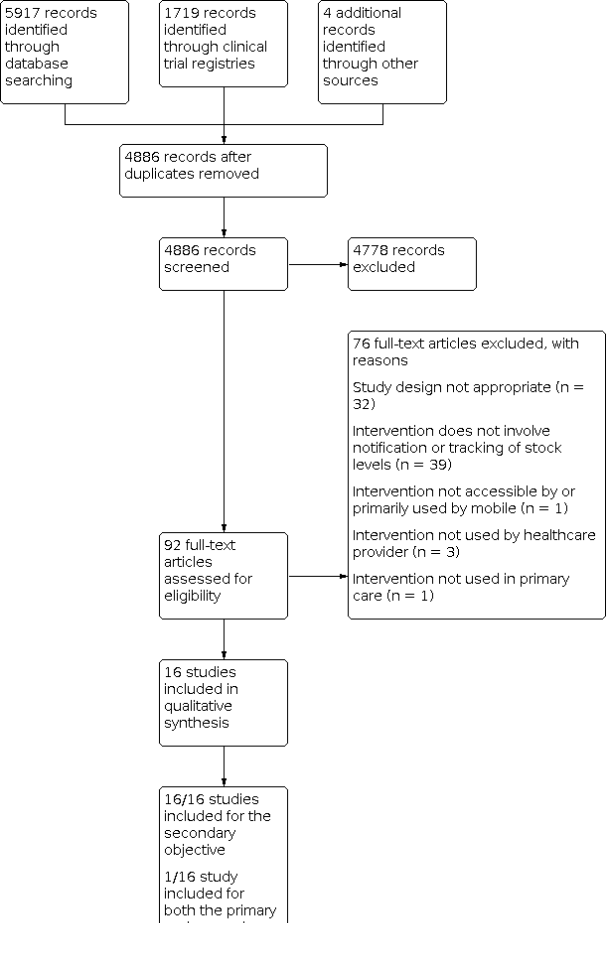
Study flow diagram

#### Included studies

##### Primary objective

We included one controlled before‐after study that met our inclusion criteria for the primary objective: to assess the effects of the intervention (Shieshia 2014). We determined that the study was a controlled before‐after study, based on our assessment of published and unpublished data. We present key characteristics of the included studies in the Characteristics of included studies table.

##### Secondary objective

We included sixteen studies that fulfilled our inclusion criteria for the review’s secondary objectives; 13 studies were peer‐reviewed articles and 5 were published reports. These described a total of 11 interventions targeted at stock notification and digital tracking of healthcare commodities. See Characteristics of included studies.

Several studies described interventions that were implemented in multiple countries. For example, one intervention called ‘SMS for Life’, described by four studies, was tested as a pilot in Tanzania (Barrington 2010; Mikkelsen‐Lopez 2014), and Kenya (Githinji 2013), and then rolled out at a larger scale across Tanzania, Kenya, Ghana, and Cameroon (WHO 2013). A few types of interventions, targeted at improving health commodity inventory, were administered under the DELIVER project. One type of intervention was described by two studies; one implemented in Zambia (USAID 2016), and the second in Tanzania, Ghana, and Liberia (USAID 2010). A study describing a second intervention was implemented in Ethiopia and Pakistan (Hara 2017). Two studies (and two additional unpublished documents) described variants of an intervention called ‘cStock’ in Malawi and Rwanda (Chandani 2017; Shieshia 2014). Two studies were implemented in Uganda (Asiimwe 2011; Namisango 2016), one in South Africa (Barron 2016), one in Ethiopia (Atnafu 2017), one in Zambia (Biemba 2017), one in India (Negandhi 2016), and one in Malawi and Ghana (Stanton 2016).

##### Interventions

The study that addressed the primary objective aimed to improve reporting, collation, and visibility of stock data. Shieshia 2014 describes two interventions, both with a common mobile web‐based reporting system called ‘cStock’. cStock is used for community–level reporting of stocks for 19 health products managed by health surveillance assistants (HSAs). In addition to cStock, one of the interventions included an enhanced management (EM) component, comprised of quality improvement teams that used data supplied by cStock, to monitor performance of the supply chain and make informed supply chain decisions (Comparison 1). The second intervention combined cStock with efficient product transport (EPT), which consisted of training all HSAs on bicycle maintenance, and providing a basic tool kit (Comparison 2).

The interventions described in all included studies for the primary and secondary objectives were targeted at notifying and managing stock levels of a range of drugs and commodities — cStock aimed to reduce stockouts of drugs for the Integrated Management of Childhood illnesses (IMCI; (Chandani 2017; Shieshia 2014)), and SMS for Life aimed to reduce stockouts of essential drugs for malaria treatment (Barrington 2010; Githinji 2013; Mikkelsen‐Lopez 2014; WHO 2013). Other interventions targeted effective vaccine management (Negandhi 2016), commodity and drug management for neglected tropical diseases (Stanton 2016), management of drugs for integrated community case management (Biemba 2017), palliative care drugs (Namisango 2016), rapid diagnostic tests and drugs for malaria (Asiimwe 2011), contraceptive products (Atnafu 2017), management of essential drugs and supplies (Barron 2016; USAID 2010; USAID 2016), and use of barcodes to improve procurement and supply coordination of health commodities (Hara 2017).

##### Outcomes

Shieshia 2014, included under the primary objective, reported on the availability of seven medicines for the treatment of childhood illnesses, timeliness of reporting on stock levels, and the acceptability of digital intervention to providers. Stock availability was measured in two ways: (1) Percentage of eligible HSA’s who reported stockout of required medicines on the day of visit; and (2) Percentage of eligible HSA’s who reported stockouts of specific medicines over the last 30 days. Timeliness of reporting on stock levels was only reported for the two intervention groups, and not for the comparison group. Acceptability of cStock was evaluated by looking at its level of routine use (e.g. HSA’s who used cStock as the primary means for ordering health products), and benefits perceived by the users. Again, these results were reported only for the two intervention groups.

All the studies included for the secondary objectives described the interventions targeted at reducing stockouts with varying levels of clarity. None of the studies aimed to formally assess the barriers and enables of implementation.

#### Excluded studies

For the primary objective, we excluded 77 articles after full‐text screening for one of the following reasons: the article did not meet the criteria for study design (N = 33); the intervention did not include a mobile device component (N = 1); the intervention did not directly target stock notification or tracking (N = 39); the intervention was not used by a healthcare provider (N = 3); or the intervention was not used in primary care (N = 1). Details of 15 potentially relevant studies, which were excluded, are provided in the 'Characteristics of excluded studies' table.

### Risk of bias in included studies

#### Risk of bias in included studies for the primary objective

For the primary objective, we have presented the risk of bias assessments for the included study in Table 5 . For Shieshia 2014, intervention groups were purposively assigned, and owing to the nature of the intervention, blinding was not possible. The study had different sample sizes for the different outcomes assessed, and more participants were included in the analyses at follow‐up compared to the baseline. Methods for random sequence generation, allocation concealment, and blinding of outcome assessment were not described in the methods. Random sequence was generated by lottery among twelve socioeconomically and topographically comparable districts. Blinding was not feasible, as the intervention involved distribution of mobile phones. Outcome data were not reported for all participants.

#### Methodological limitations of included studies for the secondary objectives

For the secondary objectives, the included studies comprised a multitude of study designs. Some were case studies that described the development and components of the intervention (Biemba 2017; Hara 2017; Stanton 2016; USAID 2010; WHO 2013). One study measured the outcome of interest before and after the intervention, without a control group (Namisango 2016). Most of the studies used operational data to describe changes in key outcomes, as the interventions were rolled out over time, and without a control group (Asiimwe 2011; Barrington 2010; Barron 2016; Chandani 2017; Githinji 2013; Mikkelsen‐Lopez 2014; Negandhi 2016; USAID 2016). All but two of the studies included for the secondary objective had significant methodological limitations – they did not include empirical data, and provided unclear descriptions of the source of the information, with limited evidence to support their findings (Atnafu 2017; Shieshia 2014). These studies described the interventions, and the conclusions were typically drawn from authors’ experiences in implementing the intervention. We have reported our assessments for each WEIRD tool item and the overall assessment for each relevant study in Table 6.

### Effects of interventions

See: Table 1; Table 2; Table 3

#### Primary objective

We included one study that met our primary objective: to assess the effects of the intervention.

In Shieshia 2014, health surveillance assistants (HSAs) used their mobile phones for community‐level reporting of data about nineteen drugs and products through a structured SMS – a system referred to as cStock. This was combined with two additional intervention components. In the enhanced management group (EM), district product availability teams were trained to use the data, monitor performance, and make informed supply chain decisions (Comparison 1). In the Efficient Product Transport (EPT) group, HSAs received a toolkit and training on bicycle maintenance (Comparison 2).

##### Comparison 1: Mobile stock notifications with enhanced management (EM) compared to standard care

See Table 1

###### Availability of commodities

We are uncertain of the effect of mobile stock notification with enhanced management on the availability of commodities (stockout of drugs in the last 30 days) compared to standard care (Shieshia 2014; very low‐certainty evidence). Stockout of drugs in the last 30 days was measured for cotrimaxazole to treat bacterial infections (Analysis 1.1), artemether‐lumefantrine to treat malaria caused by Plasmodium falciparum (Analysis 1.2; Analysis 1.3), oral rehydration drugs to treat dehydration (Analysis 1.4), and zinc to treat diarrhoea (Analysis 1.5).

###### Quality of stock management

Based on data from the intervention group only, we are uncertain of the effect of mobile stock notification with enhanced management on quality of stock management (Shieshia 2014; very low‐certainty evidence). Quality of data about stock management was assessed as the extent to which HSAs reported data about stocks that they had available. In the mobile stock notification with EM group, on average, 85% (N = 393) of the intervention group participants reported on stock levels for all the products that they managed.

###### Timeliness of stock management

Based on data from the intervention group only, we are uncertain of the effect of mobile stock notification with enhanced management on timeliness of stock management (Shieshia 2014; very low‐certainty evidence). The effect of the intervention on the timeliness of stock management was measured at the level of the health facilities. Health facilities in the stock notification with EM group took an average of 12.8 days to fill an order requested by healthcare providers.

###### Satisfaction and acceptability

Based on data from the intervention group only, we are uncertain of the effect of mobile stock notification with enhanced management on provider satisfaction (Shieshia 2014; very low‐certainty evidence). Provider satisfaction with the intervention was evaluated based on routine use. Ninety‐seven percent (N = 81) of HSAs in the stock notification with EM group reported using cStock as the primary means for ordering health products from the resupply point.

###### Resource Use

The included study did not report on the effect of the intervention on resource use.

###### Unintended consequences

The included study did not report on the effect of the intervention on unintended consequences.

##### Comparison 2: Mobile stock notification with efficient product transport (EPT) compared to standard care

See Table 2

###### Availability of commodities

We are uncertain of the effect of mobile stock notification with EPT on the availability of commodities (stockout of drugs in the last 30 days) compared to standard care (Shieshia 2014; very low‐certainty evidence) stockout of drugs in the last 30 days was measured for cotrimaxazole to treat bacterial infections (Analysis 2.1), artemether‐lumefantrine to treat malaria caused by Plasmodium falciparum (Analysis 2.2; Analysis 2.3), oral rehydration drugs to treat dehydration (Analysis 2.4), and zinc to treat diarrhoea (Analysis 2.5).

###### Quality of stock management

Based on data from the intervention group only, we are uncertain of the effect of mobile stock notification with EPT on the quality of stock management (Shieshia 2014; very low‐certainty evidence). Quality of data was measured as the extent to which HSAs reported data about stocks that they had available. In the mobile stock notification with EPT group, on average, 65% (N = 253) of the HSAs reported on stock levels for all the products that they managed.

###### Timeliness of stock management

Based on data from the intervention group only, we are uncertain of the effect of mobile stock notification with EPT on the timeliness of stock management (Shieshia 2014; very low‐certainty evidence). Effect of the intervention on the timeliness of stock management was measured at the level of the health facilities. Health facilities in the stock notification with EPT group took an average of 26 days to fill an order requested by healthcare providers.

###### Satisfaction and Acceptability

Based on data from the intervention group only, we are uncertain of the effect of mobile stock notification with EPT on provider satisfaction (Shieshia 2014; very low‐certainty evidence). Provider satisfaction with the intervention was evaluated based on routine use. Ninety‐one percent (N = 78) of the HSAs in the stock notification with EPT group reported using cStock as the primary means for ordering health products from the resupply point.

###### Resource Use

The included study did not report on the effect of the intervention on resource use.

###### Unintended consequences

The included study did not report on the effect of the intervention on unintended consequences.

#### Secondary objectives

##### Current use of mobile strategies to improve reporting and digital tracking of health commodities

We included 16 studies that met the first of our secondary objectives; to describe how these types of mobile strategies are currently being used. These studies described eleven different interventions, all of which aimed to reduce stockouts. See Study characteristics for the secondary objectives in Characteristics of included studies.

Figure 2 summarises the key intervention components that we identified in eight of these eleven interventions. The overarching purpose of each of these interventions was to make data about stock levels and potential stockouts of commodities visible to managers, who could then take corrective action to address them. Each of these interventions required the use of a mobile device by healthcare workers, either to report stock levels as a text message (Asiimwe 2011; Barrington 2010; Chandani 2017; Githinji 2013; Mikkelsen‐Lopez 2014; Shieshia 2014; WHO 2013), or to collect data about stock levels, using a digital data collection form or an app (Atnafu 2017; Biemba 2017; Negandhi 2016; Stanton 2016; USAID 2010; USAID 2016). If a text message was used to report stock levels using the healthcare worker’s personal mobile phone, the messages were sent to a short code that was free of cost to the health care worker (Asiimwe 2011; Barrington 2010; Chandani 2017; Githinji 2013; Mikkelsen‐Lopez 2014; Shieshia 2014). These data were collected and aggregated on a central server, and made available on a webpage as an electronic database (Stanton 2016; USAID 2010; USAID 2016), in the form of an interactive dashboard (Asiimwe 2011; Barrington 2010; Chandani 2017; Githinji 2013; Mikkelsen‐Lopez 2014; Negandhi 2016; Shieshia 2014; WHO 2013), or as routine reports (Atnafu 2017; Biemba 2017). It was intended that this webpage be monitored by a higher level (e.g. district level) health official, who was responsible for taking corrective action to address extant or expected stock or commodity shortages. Some authors reported instituting active measures to ensure that the online data were used and responded to in a timely fashion. Shieshia 2014 described how additional staff, with clearly defined roles, were hired to monitor the data and respond to them. In ‘SMS for Life’, weekly summary reports were provided to district medical officers and pharmacists in addition to the dashboards, to support data use (Barrington 2010; Githinji 2013; Mikkelsen‐Lopez 2014; WHO 2013).

**2:**
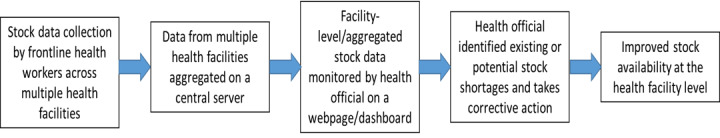
Common key intervention components of interventions targeted at reducing stock‐outs

The following features were salient to some of the eight interventions described in Figure 2.

- **Reminders to healthcare workers to send reports.** Three of the eight interventions described a feature that sent reminders to healthcare workers to send their weekly stock‐level reports (Barrington 2010; Githinji 2013; Mikkelsen‐Lopez 2014; Shieshia 2014).
- **Incentives to health workers.** A few studies described incorporating performance‐based incentives for the healthcare workers to send timely stock reports. For the implementation of SMS for Life, in Tanzania, Mikkelsen‐Lopez 2014 reported that if the healthcare worker sent a report on time, they received a phone credit of TZS 1000, or USD 0.70, for personal use. In Kenya, 50 Kenyan Shillings, or USD 0.6 worth of airtime was transferred to healthcare workers who sent timely stock reports (Githinji 2013).
- **Two‐way digital communication.** In some of the interventions, there were checks in place to ensure that the messages sent by the healthcare workers were free of error. In the case of an error in the format of the message, the health worker would receive an error message, advising them to correct their message (Asiimwe 2011; Barrington 2010; Githinji 2013). When there were no errors, the healthcare worker received a confirmation message, stating that their weekly stock report had been received (Asiimwe 2011; Barrington 2010; Chandani 2017; Githinji 2013; Shieshia 2014).
- **Multi‐faceted interventions.** Some of the interventions targeted at reducing stockouts were part of a broader multi‐faceted intervention. In addition to digital data reporting, aggregation, and visualisation, cStock invested in training district and central‐level staff to use computers to access web‐based dashboards for reporting, and quality improvement teams to use the data supplied by cStock to monitor performance of the supply chain and make informed supply‐chain decisions (Shieshia 2014). The LMS Suite, described by Stanton 2016, comprised three tools, each targeted at different aspects of managing cases of lymphatic filariasis, including: (1) 'MeasureSMS‐MDA' to support text message mass drug administration (MDA) and reporting of cases that had been treated for elephantiasis using appropriate anti‐parasitic medicines; (2) 'Measure‐SMS‐morbidity' to report on new cases of elephantiasis and their demographic information; and (3) 'Ly‐MSS' lymphedema management support system aimed to maintain the supply chain of care packages (such as washbasins, towels, soaps, antibacterial cream).

Three of the eleven interventions did not include the key components described in Figure 2. One of these interventions was MomConnect, described by Barron 2016. MomConnect is a national‐level service in South Africa that is targeted at connecting pregnant women to health services. Registration on MomConnect puts demographic and pregnancy‐specific information about women on an electronic database. Women then receive free informational messages, as per their stage of pregnancy, till their infant is one year old. Using this service, women can also interact with a help desk, located in the South African Department of Health (DoH) to (1) answer a brief survey about the quality of services received at the health facility, using unstructured supplementary service data (USSD) at no cost to them; (2) ask any question related to their pregnancy, using a text message; and (3) log a complaint or a compliment, using a free of cost text message. The complaints could be about commodity or medicine shortages during their last visit to the health facility, and are routinely monitored and addressed by the help desk.

The second intervention was the palliative care management software reported in Namisango 2016. This intervention included data collection, an electronic health record for the patient, and functions for supply chain management and provider work‐planning and scheduling. The health care provider could use a tablet computer to check their mobile application screen for which drugs were available in the pharmacy before prescribing the drug. Providers could enter patient and pharmacy data on the application. These data were linked to the database available to the pharmacist, who was responsible for tracking inventory based on the number of prescriptions written for a certain drug.

The third intervention was the use of bar codes to record and track health products reported in Hara 2017. They used an automatic identification and data capture system (AIDC), using barcodes based on global standard to improve end‐to‐end supply chain visibility of health commodities. The bar codes uniquely identified each health product and were linked to the batch and serial numbers of the products, and expiration dates. They described a process in which a smartphone application was used, in Ethiopia, to scan bar codes using the mobile phone’s camera, and push the captured data to a central logistics management software. This made real time tracking of commodities feasible, and built efficiencies in the system to streamline the availability of products, where needed.

##### Factors influencing implementation

We also used these 16 studies to address the second of our secondary objectives: to identify factors influencing the implementation of mobile interventions targeted at reducing stockouts of health commodities. All but one of these studies lacked empirical data, and clear descriptions of the source of the information, resulting in limited evidence to support the findings (Shieshia 2014). The studies described the interventions used, and the conclusions were typically drawn from the study authors’ experiences in implementing the intervention. We considered these limitations in our GRADE‐CERQual assessment of confidence in our findings. We also noted in the results that the perspective of the findings is, in general, that of the study authors.

The study authors referred to several factors that may influence the implementation, uptake, or efficient use of interventions targeted at improving stock management. In Figure 3, we grouped these factors under three categories: macro‐level factors that constitute a supporting ecosystem; programmatic factors associated with implementation; and factors directly pertaining to the technological component of the intervention. We also used these as a summary of findings for the secondary objective in Table 3.

**3:**
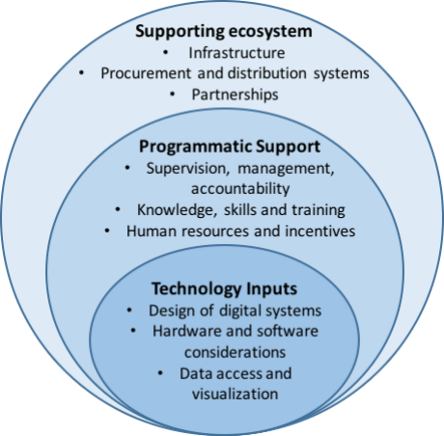
Enablers and barriers of implementation of interventions targeted at commodity stock‐outs

###### Supporting ecosystem

####### a. Infrastructure

Study authors identified several infrastructural issues that they suggested directly influence the implementation of mobile interventions, targeted at improving notification and tracking of commodities. These included problems with poor access to electricity, and poor or non‐existent internet connectivity, leading to challenges in charging phones, uploading and transmitting data, and loss of data (Asiimwe 2011; Atnafu 2017; Negandhi 2016; Shieshia 2014; Stanton 2016; USAID 2010; moderate‐certainty evidence; Finding 1; Table 3).

About one‐fifth of all participants in one study reported challenges in sending text messages due to poor network (Shieshia 2014). Authors identified how some of these issues could be mitigated: using solar energy packs for charging phones (Namisango 2016), developing systems for working offline during internet outages (Namisango 2016), training health workers to resend their reports the next day, in case network coverage is not available initially (Asiimwe 2011), and taking steps upfront to ensure that network coverage is available within a few hours of the health facilities where the intervention is being implemented, so that reports can be submitted when health workers reach areas with better connectivity (Barrington 2010; Biemba 2017). However, health facilities that are responsible for receiving patient referral data from the community need to have regular connectivity (Biemba 2017).

####### b. Procurement and distribution systems

Study authors were concerned that digital stock notification systems used at the facility level could not mitigate several broader health system problems, including an underlying lack of stock at the national or district level, and a mismatch between national ordering routines and local needs (low‐certainty evidence; Finding 2; Table 3).

Nationally instituted procurement and distribution systems (Chandani 2017), and inventory management information systems were identified as important considerations for implementation (Hara 2017). For example, the procurement of health commodities is influenced by donor policies. Mikkelsen‐Lopez 2014 reported that during the implementation of their intervention ‘SMS for Life’, the Tanzanian government had reduced its malaria budget significantly, because over half the malaria drugs were provided by donors. However, a two‐year delay in one of the donor funding cycles resulted in a national, critical, unanticipated drug shortage. Shortages at the national and regional levels cannot be mitigated by any corrective action taken at the district level. Such shortages also result in poor morale of managers (Githinji 2013).

Poor reconciliation between national and district drug procurement systems and the medicine ordering system makes it challenging to order the correct amount of drugs (Mikkelsen‐Lopez 2014). For example, in Tanzania, health facility drug orders are made quarterly, based on the patterns of the previous quarter. However, this does not account for the seasonality of diseases like malaria (Mikkelsen‐Lopez 2014).

####### c. Partnerships

Study authors described how programmes could benefit from strong partnerships, including with local authorities who could provide training for healthcare staff and district managers; with local telecommunications companies who could offer good rates for SMS transfer; with technical system providers who could support the development of the system; and with non‐governmental organizations (NGOs) who could support the roll‐out and training of the intervention (WHO 2013; very low‐certainty evidence; Finding 3; Table 3).

###### Programmatic Support

####### a. Data use, supervision, and management

Study authors suggested that the availability and use of data on stock levels at all levels of the health system allowed healthcare officials to respond to anticipated shortages (low‐certainty evidence; Finding 4; Table 3). The use of digital data on stock availability was one of the key features of all the interventions identified by this review. Barron and colleagues suggested that data visibility and use are important at all levels of the health system, from the district to the national level (Barron 2016). Quick sharing of data across health workers and facilities was found useful by healthcare workers in Ghana and Malawi (Stanton 2016).

Shieshia and colleagues reported that in addition to data dashboards, comprising of information on stock levels, being available online in a digital format, stock reports were printed routinely at the district level. These stock reports and health facility performance and challenges were discussed at district‐level meetings, allowing health workers across levels of the health system to become aware of the stock management procedures (Shieshia 2014). While most studies found the availability of stock data across levels of the health system to be useful, authors of one study highlighted a risk of making data accessible in real time across multiple levels of management. For instance, authors of a study in Uganda reported that district health officials are typically used to having greater control over the data that they report to the national level. With a digital system to track and report stock levels, data becomes simultaneously available to the district and national‐level stakeholders, and takes the opportunity away from the district officials to contextualise the data or explain shortcomings (Asiimwe 2011).

Authors emphasised the role of supportive supervision and responsive management for effective adoption of a digital system (moderate‐certainty evidence; Finding 5; Table 3). Adequate supervision of the stock notification systems and associated data were identified as vital to successful implementation (Barrington 2010; Negandhi 2016; Shieshia 2014). For example, supervision of district level staff was needed to ensure that the data on stock levels were used, and appropriate corrective action was taken in a timely manner (Shieshia 2014). Routine visits and meetings of supervisors and healthcare workers can facilitate problem‐solving, and ensure timely communication to discuss any resupply procedures (Chandani 2017). In structuring the management, programmes should consider that health workers must be motivated to report the data, and supervisors must be motivated to use the data (Chandani 2017). Some study authors highlighted the importance of having well defined roles and responsibilities for the management staff (Asiimwe 2011; Barrington 2010; Shieshia 2014), and strict timelines for the roll‐out of the intervention, to further accountability (Barrington 2010). In some areas in Uganda, district health officials, who were expected to monitor stock levels and respond to them, only became involved in an ad‐hoc manner. The authors reported that this could be circumvented by having clearer roles defined by the Ministry of Health (Asiimwe 2011).

####### b. Knowledge, skills, and training

Some of these factors associated with implementation were tied to healthcare workers’ and staff members’ knowledge and skills, including the extent to which they were familiar with smartphones, comfortable using mobile data services (Stanton 2016), and the extent to which they were given adequate training in using the digital system (Negandhi 2016; Shieshia 2014; moderate‐certainty evidence; Finding 6; Table 3).

Some authors highlighted the value of training to improve the uptake of the digital intervention, including training of the frontline health workers, such as Health Surveillance Assistants (HSA), who may be involved in sending stock reports (Shieshia 2014), training of support staff, such as cold chain technicians, who might be involved with stock management (Negandhi 2016), and facility workers, who count the stock levels (Barrington 2010; Githinji 2013). Authors suggested that staff should be trained in the composition and use of text messages (Asiimwe 2011; Barrington 2010; Githinji 2013). Training should also be provided to management staff at the national and district levels in the use of data and online dashboards (Barrington 2010; Githinji 2013).

####### c. Human resources and incentives

Authors identified that to support successful implementation of a digital intervention, it was important to have technical programming expertise available to develop and install the digital programme, and to maintain the system on an ongoing basis (Asiimwe 2011; USAID 2010). Asiimwe 2011 suggested that locally available expertise in software programming was important to responsively develop and test the mobile applications. Having ongoing technology support was important to address software bugs and other problems once the system was piloted (USAID 2010; low‐certainty evidence; Finding 7; Table 3).

As discussed earlier, several studies offered performance‐based incentives of mobile phone credit to health workers, for timely reporting. While the value of such performance‐based incentives was not formally assessed, one study author reported that they found that incentives in the form of airtime credit to healthcare workers was helpful in encouraging timely SMS reporting of stock levels (Barrington 2010; low‐certainty evidence; Finding 8; Table 3).

###### Technology inputs

####### a. Design of the digital systems

Several factors affecting implementation were tied to the design of the digital system, including the extent to which the systems were user‐friendly, with easy‐to‐use interfaces, and built with user participation (Namisango 2016; Negandhi 2016; Shieshia 2014), and the extent to which they were aligned with the country's existing health information reporting systems (Shieshia 2014). Two study authors emphasised the importance of iteratively designing the platform with user feedback and input to improve the acceptability and adoption of the digital intervention (Namisango 2016; Shieshia 2014; moderate‐certainty evidence; Finding 9; Table 3).

####### b. Digital hardware and software

Study authors considered the use of basic mobile phones or personal phones by health workers to reduce challenges with data coverage and expense, and support easier adoption of the intervention due to familiarity with the phones (low‐certainty evidence; Finding 10; Table 3). One study author suggested that programmes might consider using basic phones in lieu of android phones, as data network coverage is limited in remote locations, and data packages are prohibitively expensive (Stanton 2016). Another author suggested that having health workers use their personal mobile phones mitigates problems with phone maintenance, familiarity, and issues of ownership (Barrington 2010). Managers might be provided with a Blackberry or a similar device, so they can access dashboards, especially in places where desktop computer access is limited (Barrington 2010).

Study authors highlighted software features, such as ability to capture images, map geographic features, support two‐way communication, toll‐free text messaging, and interoperability (low‐certainty evidence; Finding 11; Table 3). Software that had multiple features, such as the ability to capture images and map geographical locations, was amenable to programming, and could be used for different programmes was preferable (Negandhi 2016). Having a function for two‐way communication with the healthcare workers, either to confirm receipt of their stock reports, or to send them updates on stock availability, helped them to take necessary action, and supported morale (Shieshia 2014). One author emphasised the value of a toll‐free number for text messaging, so that health workers were not deterred by anticipated costs in sending text message on stock‐level updates (Barrington 2010). Negandhi and colleagues identified interoperability of the stock management systems as important for success (Negandhi 2016). The authors suggested that logistics management systems should be linked to health management systems, so that linkages could be made between supply and demand, which should in turn, could reduce waste.

####### c. Data visualisation

Several factors that influenced implementation were tied to the design of the dashboards, and data visualisation. Authors emphasised that healthcare mangers should have access to data in an easy‐to‐use format (Shieshia 2014), with an effective display of data using factsheets, and graphical and tabular illustrations (Negandhi 2016; moderate‐certainty evidence; Finding 12; Table 3).

In order to accommodate this, Shieshia 2014 reported redesigning the dashboards several months after the system was set up, so that the users had a better understanding of the metrics and visuals, and could incorporate their experiences interacting with the system into the redesign. The management of data should be detail‐oriented, with regular reviews of the database (USAID 2010). To facilitate visualisation of data, healthcare personnel at other levels of the healthcare system also need access to functioning smart phones, laptops, or desktop computers (Biemba 2017).

## Discussion

### Summary of main results

Our review provides limited evidence on the primary objective, to assess the effect of tracking health commodity inventory and notifying stock levels via mobile devices on improvements in availability of commodities, quality of data about stock management, timeliness of stock‐level reporting, and provider acceptability. We identified one study, conducted in Malawi, that used a before‐after study design to answer these questions (Shieshia 2014). However, we are uncertain of the effect of these interventions on the outcomes of interest, because we assessed the certainty of this evidence as very low.

For the secondary objectives, we included 16 studies that described a total of eleven interventions. All studies were conducted in Africa (Tanzania, Kenya, Malawi, Ghana, Ethiopia, Cameroon, Zambia, Liberia, Uganda, South Africa, and Rwanda) and Asia (Pakistan and India). Most of the interventions aimed to make data about stock levels and potential stockouts visible to managers, who could then take corrective action to address them. We identified several factors that may influence the implementation of stock notification and tracking via mobile device. These included challenges tied to infrastructural issues, such as poor access to electricity or internet, and broader health systems issues, such as drug shortages at the national level, which could not be mitigated by interventions at the primary healthcare level. Several factors were identified as important, including strong partnerships with local authorities, telecommunication companies, technical system providers, and non‐governmental organizations; availability of stock‐level data at all levels of the health system; the role of supportive supervision and responsive management; familiarity and training of health workers in the use of the digital devices; availability of technical programming expertise for initial development and ongoing maintenance; incentives, such as phone credit, to support regular use of the system; easy‐to‐use systems built with user participation; use of basic or personal mobile phones to support easier adoption; consideration for software features, such as two‐way communication; and data availability in an easy‐to‐use format, such as an interactive dashboard.

### Overall completeness and applicability of evidence

We identified insufficient high‐quality studies to address the primary objective of the review. Evidence was insufficient to recommend the use of mobile tools to track health commodity inventory and stock notification. We did not identify any data on use of resources for such interventions, or unintended consequences. Despite the proliferation of large scale, mobile‐based interventions to support stock notification and management, we did not identify any ongoing studies to address questions on the effectiveness of such interventions.

For the secondary study objective, the studies that described mobile interventions targeted at stock management had some common features, involving timely collection of stock data, visibility of stock data at different levels of the health system, and use and responsiveness to these data. Several implementation challenges that were identified by this review are consistent with the global evidence that points to general considerations for the implementation of digital interventions, including problems with network connectivity, access to electricity, device usability, and access to health worker training. Several 'best practices' were identified, based on the experiences of the study authors in implementing the interventions. Given the lack of empirical data from which these conclusions were drawn, and the high level of contextual and infrastructural variability within which such interventions might be implemented, these findings have limited external validity, and should be cautiously interpreted.

### Quality of the evidence

We used the GRADE methodology to assess the quality of evidence for the primary objective, and GRADE‐CERQual to assess the quality of evidence for the secondary objective. The quality of evidence relating to all five outcomes under the primary objective were downgraded two levels, due to very serious risk of bias concerns, and one level for imprecision, due to a small sample size. Outcomes of quality and timeliness of stock management, and satisfaction and acceptability of the intervention by providers, were downgraded to very low‐quality evidence, as these were based on data from the intervention group only.

All but one study included for the secondary objective had significant methodological limitations – they did not include empirical data, had unclear descriptions of the source of the information, resulting in limited evidence to support the findings (Shieshia 2014). These studies described the interventions, and the conclusions were typically drawn from authors’ experiences in implementing the intervention. Our confidence in the evidence for the secondary objective was typically downgraded due to methodological limitations of the studies, and adequacy of the findings, owing to a small number of studies contributing to specific findings.

### Potential biases in the review process

We do not believe that the potential for bias in the review process for the primary objective was high. The authors meticulously followed the protocol.  Where necessary, we attempted to contact the study authors to request missing relevant information.

For the secondary objective, while we followed the study protocol, the inclusion criteria were broadly defined. This could have resulted in the omission of certain articles and reports, especially if these were not published on any of the search engines that we outlined in our approach. In some cases, where reporting on the details of the intervention or factors affecting its implementation was unclear or incompletely reported, the review authors attempted to infer relevant findings from the study authors’ presented opinions.

The review team represents diverse professional backgrounds, which could have influence our input in conducting this review. Three of the review authors (SA, TT, GM) have been closely involved with the development and deployment of digital interventions in low‐ and middle‐income countries, and have experience in conducting primary research to evaluate digital health interventions. One review author (SA) is a co‐author on the study included in the primary objective. While these experiences provided us with a platform for understanding the complexities and nuances of evaluating such interventions, they may also have influenced our analyses of the studies included in this review. We tried to moderate this influence by working closely with other review authors. SA questioned the weight she attributed to certain data that resonated with her experiences, and ensured that all data were equally weighted in the final set of findings. Other members of the review team were called upon to verify the findings and ensure that they were supported by the data. As is standard practice in qualitative research, two authors conducted the GRADE‐CERQual assessment.

### Agreements and disagreements with other studies or reviews

To our knowledge, this is the first systematic review of mobile phone‐based interventions for tracking health commodity inventory and stock notification, and trying to understand factors that affect implementation of interventions targeted at improving stock availability. One literature review assessed the potential impact of mobile‐based interventions on drug supply chain and stock management as one of several outcomes (Aranda‐Jan 2014). The review narratively summarised results from two studies that were excluded from the primary objective, as they did not meet the study design inclusion criteria (Barrington 2010; Githinji 2013). It concluded that evidence was insufficient to assess the impact of mobile devices on drug stock management.

## Authors' conclusions

**Implications for practice**

Below are a set of questions that may help health system or programme managers when implementing or planning tracking health commodity inventory and notifying stock levels via mobile devices.

**1. Have you considered the availability of necessary infrastructure?**

- Do health workers have reliable access to electricity and internet connectivity?
- Where network access is a challenge, are there systems in place so that staff can work offline until connectivity is restored?
- Have you considered whether health workers might prefer to use basic or simple mobile phones, or their own personal phones, rather than smart phones, for instance because their own phones might be cheaper and easier to use?
- Do you have reliable access to the medicines and supplies that local health facilities need? Are systems in place for regular procurement of medicines at the national and sub‐national level, so that supplies can be made available when requested through digital notification systems?

**2. Have you taken the needs and view of users into account when developing, planning, and implementing the use of mobile devices for stock notification and tracking?**

Have you considered the type and format of data and information that should be presented on a dashboard?

- Will you involve users in an iterative design process, with the system evolving as the needs of users and the health system become clear?
- Have you selected technology that is appropriate for your setting’s data network coverage, data needs, and local capacity for maintenance?
- Have you put in place mechanisms to select software that aligns with programme needs for specific functions, such as capturing images, mapping geographical locations, and two‐way communication?

**3. Have you considered how to work with key partners and how to share data?**

- Have you considered partnering with local authorities to facilitate effective implementation? This may include partnerships with the government, local telecommunication companies, technical systems providers, or non‐governmental organisations (NGO).
- Have you ensured that data are available at all levels of the health system? Would developing digital dashboards help in making data available to managers at district, regional, and national levels? If developing dashboards is not feasible, is it possible to develop a paper‐based system for sharing stock availability reports with district and national levels?

**4. Is there a plan for addressing training and support needs?**

- Have you ensured that health facility staff have adequate training in the use of the digital system, and where necessary, in the use of any equipment, such as smart phones?
- Do health facility staff have sufficient mobile phone credit to support timely reporting of stock data and adoption of the system?
- Do you have the technical programming expertise that is needed to develop, install, and maintain the system on an ongoing basis?
- Have you ensured that health facility staff have access to supportive supervision and responsive management structures?

These questions were drawn, based on this review. They also align with similar implications for practice identified in a linked review on health workers’ perceptions and experiences of using mHealth technologies to deliver primary healthcare services (Odendaal 2020).

**Implications for research**

1. Further, well conducted, comparative evaluations are needed to robustly establish the effects of stock notification and commodity management via mobile devices on improved availability, improve timeliness of stock availability (average time between sending an order request and receiving health products), and reduced stockout of commodities at the point of care. Given the practical challenges in randomising such systems‐level interventions, researchers may consider alternate study designs, such as controlled before‐after studies with at least two intervention and control sites, and interrupted time series studies with at least three data points before and after the intervention.
2. Interventions targeted at improvements in stock management have a large amount of variability in core intervention components. Therefore, it is important that research studies describe interventions in sufficient detail that readers can discern the core components.
3. Currently, there is no standardisation of outcomes related to measurement of stockouts and other outcomes of interest in this review. In the studies included in this review, stockout outcomes were reported in three different ways: the proportion of health workers reporting stockout of specific drugs on the day of the interview; the proportion of health workers reporting stockout of specific drugs in the last 30 days; and the proportion of women of reproductive ages who reported a stockout (of preferred contraceptive) at the health facility (over an unspecified time period). Consistency in measurement of outcomes, and use of standardised metrics, where possible, can help facilitate comparability, pooling, and meta‐analysis of the research findings.
4. Comparative evaluations should be accompanied by process evaluations to enhance understanding of the mechanisms and contexts within which different mobile‐based stock notification and commodity management interventions work well, and the views and experiences of those using these systems. Understanding the conditions under which such systems adequately operate is valuable. For example, the review shows that misalignment of national stock ordering systems and local needs limits the effectiveness of such digital systems. These process evaluations need to be well conducted, and should report their methods clearly.
5. Studies are needed of how different mobile‐based stock notification and commodity management systems can be sustainably adopted and used. This review suggests that the use of incentives, such as mobile phone airtime credit, may be considered, and it would be valuable to test empirically the effects of this and other incentives on the adoption and long‐term use of such systems.
6. The cost‐effectiveness of different mobile‐based stock notification and commodity management systems, compared to paper‐based stock‐management systems, should be assessed.
7. While there are certain common implementation considerations for mobile‐based stock notification and commodity management systems, factors influencing implementation may vary by the exact type of intervention. Research studies should identify specific factors influencing implementation by intervention characteristics.

## History

Protocol first published: Issue 1, 2018 Review first published: Issue 10, 2020

## Notes

The text under the Methods section is based on standard text and guidance provided by Cochrane Effective Practice and Organisation of Care (EPOC).

## Appendices

### Appendix 1. Search strategies

**CENTRAL; 2019, issue 7, in the Cochrane Library (searched 07 August 2019)**

**Table:**

ID	Search	Hits
#1	MeSH descriptor: [Cell Phone] this term only	620
#2	MeSH descriptor: [Smartphone] this term only	250
#3	MeSH descriptor: [MP3‐Player] this term only	21
#4	MeSH descriptor: [Computers, Handheld] this term only	239
#5	((cell* or mobile*) near/1 (phone* or telephone* or technolog* or device*)):ti,ab,kw	3495
#6	(handheld or hand‐held):ti,ab,kw	1984
#7	(smartphone* or smart‐phone* or cellphone* or mobiles):ti,ab,kw	2603
#8	((personal near/1 digital) or (PDA near/3 (device* or assistant*)) or MP3 player* or MP4 player*):ti,ab,kw	286
#9	(samsung or nokia):ti,ab,kw	115
#10	(windows near/3 (mobile* or phone*)):ti,ab,kw	4
#11	android:ti,ab,kw	478
#12	(ipad* or i‐pad* or ipod* or i‐pod* or iphone* or i‐phone*):ti,ab,kw	771
#13	(tablet* near/3 (device* or computer*)):ti,ab,kw	609
#14	MeSH descriptor: [Telemedicine] this term only	1741
#15	MeSH descriptor: [Videoconferencing] this term only	160
#16	MeSH descriptor: [Webcasts as Topic] this term only	21
#17	MeSH descriptor: [Text Messaging] this term only	664
#18	MeSH descriptor: [Telenursing] this term only	28
#19	(mhealth or m‐health or "mobile health" or ehealth or e‐health or "electronic health"):ti,ab,kw	3598
#20	(telemedicine or tele‐medicine or telehealth or tele‐health or telecare or tele‐care or telenursing or tele‐nursing or telepsychiatry or tele‐psychiatry or telemonitor* or tele‐monitor* or teleconsult* or tele‐consult* or telecounsel* or tele‐counsel* or telecoach* or tele‐coach*):ti,ab,kw	5019
#21	(videoconferenc* or video‐conferenc* or webcast* or web‐cast*):ti,ab,kw	664
#22	(((text* or short or voice or multimedia or multi‐media or electronic or instant) near/1 messag*) or instant messenger) .ti,ab,kw	53
#23	(texting or texted or texter* or ((sms or mms) near (service* or messag*)) or interactive voice response* or IVR or voice call* or callback* or voice over internet or VOIP):ti,ab,kw	2361
#24	(Facebook or Twitter or Whatsapp* or Skyp* or YouTube or "You Tube" or Google Hangout*):ti,ab,kw	762
#25	MeSH descriptor: [Mobile Applications] this term only	420
#26	"mobile app*":ti,ab,kw	393
#27	MeSH descriptor: [Social Media] this term only	108
#28	(social near (media or network*)):ti,ab,kw	2162
#29	MeSH descriptor: [Reminder Systems] this term only	857
#30	(remind* near/3 (text* or system* or messag*)):ti,ab,kw	1825
#31	MeSH descriptor: [Electronic Mail] this term only	304
#32	(electronic mail* or email* or e‐mail or webmail):ti,ab,kw	4063
#33	MeSH descriptor: [Medical Informatics] this term only	72
#34	MeSH descriptor: [Medical Informatics Applications] this term only	23
#35	MeSH descriptor: [Nursing Informatics] this term only	10
#36	MeSH descriptor: [Public Health Informatics] this term only	1
#37	((medical or clinical or health or healthcare or nurs*) near/3 informatics):ti,ab,kw	311
#38	MeSH descriptor: [Multimedia] this term only	212
#39	MeSH descriptor: [Hypermedia] this term only	8
#40	MeSH descriptor: [Blogging] this term only	13
#41	(multimedia or multi‐media or hypermedia or hyper‐media or blog* or vlog* or weblog* or web‐log*):ti,ab,kw	1227
#42	MeSH descriptor: [Interactive Tutorial] this term only	0
#43	MeSH descriptor: [Computer‐Assisted Instruction] this term only	1179
#44	((interactive or computer‐assisted) near/1 (tutor* or technolog* or learn* or instruct* or software or communication)):ti,ab,kw	1442
#45	{or #1‐#44}	26519
#46	MeSH descriptor: [Vaccines] explode all trees and with qualifier(s): [economics ‐ EC, standards ‐ ST, supply & distribution ‐ SD]	224
#47	MeSH descriptor: [Equipment and Supplies] explode all trees and with qualifier(s): [economics ‐ EC, standards ‐ ST, statistics & numerical data ‐ SN, supply & distribution ‐ SD]	3563
#48	MeSH descriptor: [Therapeutic Uses] explode all trees	159576
#49	MeSH descriptor: [Pharmaceutical Preparations] explode all trees and with qualifier(s): [economics ‐ EC, standards ‐ ST, supply & distribution ‐ SD]	262
#50	MeSH descriptor: [Pharmaceutical Services] this term only	148
#51	MeSH descriptor: [Community Pharmacy Services] this term only	241
#52	MeSH descriptor: [Drug Information Services] this term only	43
#53	MeSH descriptor: [Pharmaceutical Services, Online] this term only	0
#54	MeSH descriptor: [Pharmacy Service, Hospital] this term only	135
#55	MeSH descriptor: [Hospital Distribution Systems] this term only	1
#56	MeSH descriptor: [Materials Management, Hospital] this term only	2
#57	MeSH descriptor: [Inventories, Hospital] this term only	0
#58	MeSH descriptor: [Medication Systems, Hospital] this term only	29
#59	MeSH descriptor: [Product Line Management] this term only	0
#60	((commodit* or consumable* or stock or stocks or inventor* or supply or supplies) near/3 (level* or notif* or track* or count* or report* or chain or out or outs or manag* or order* or logistic* or system or systems or shortage* or manag* or monitor* or maintain* or maintenance or audit or auditing)):ti,ab,kw	929
#61	((health or medical or medicines or vaccine* or drug or drugs or laborator* or diagnos*) near/3 (product* or supply or supplies or consumable* or commodit* or stock or stocks or stockout* or "stock out*" or shortage*)):ti,ab,kw	4708
#62	MeSH descriptor: [Health Resources] this term only	385
#63	MeSH descriptor: [Clinical Laboratory Information Systems] this term only	8
#64	MeSH descriptor: [Clinical Pharmacy Information Systems] this term only	21
#65	MeSH descriptor: [Database Management Systems] this term only	15
#66	MeSH descriptor: [Hospital Information Systems] this term only	42
#67	MeSH descriptor: [Ambulatory Care Information Systems] this term only	25
#68	MeSH descriptor: [Medication Systems, Hospital] this term only	29
#69	MeSH descriptor: [Pharmacy Administration] this term only	2
#70	MeSH descriptor: [Drug Utilization] explode all trees	487
#71	MeSH descriptor: [Drug Storage] this term only	70
#72	{or #46‐#71}	168632
#73	MeSH descriptor: [Community Mental Health Services] this term only	693
#74	MeSH descriptor: [Family Practice] this term only	1965
#75	MeSH descriptor: [Home Care Services] this term only	1726
#76	MeSH descriptor: [Physicians, Family] this term only	444
#77	MeSH descriptor: [Physicians, Primary Care] this term only	144
#78	MeSH descriptor: [Community Health Services] this term only	949
#79	MeSH descriptor: [Community Pharmacy Services] this term only	241
#80	MeSH descriptor: [Health Facilities] this term only	75
#81	MeSH descriptor: [Health Facility Administration] this term only	0
#82	MeSH descriptor: [Community Health Nursing] explode all trees	341
#83	MeSH descriptor: [Home Health Nursing] this term only	8
#84	MeSH descriptor: [Family Nursing] this term only	36
#85	MeSH descriptor: [Community Health Workers] this term only	426
#86	MeSH descriptor: [Preventive Health Services] this term only	468
#87	MeSH descriptor: [Primary Health Care] this term only	3821
#88	MeSH descriptor: [Primary Prevention] this term only	816
#89	MeSH descriptor: [Public Health] this term only	216
#90	MeSH descriptor: [Rural Health Services] this term only	331
#91	("primary care" or "general practi*" or "primary health" or "community mental health*" or "family practice" or "family medicine" or "family doctor*" or "family physician*" or "home care" or "home based" or "home health*" or "community health*" or "community nurs*" or "health visit*" or "community pharmac*" or "preventive care" or "prevention program*" or "preventive service*" or "preventive health" or "primary prevention" or "public health" or "rural health" or "health promotion" or "health facilit*"):ti,ab,kw	56066
#92	{or #73‐#91}	56439
#93	#45 and #72 and #92	336

**MEDLINE Ovid and Epub Ahead of Print, In‐Process & Other Non‐Indexed Citations and Daily (1946 to 07 August 2019)**  

**Table:**

**#**	**Searches**	**Results**
1	Community Mental Health Services/	18191
2	Family Practice/	64597
3	Home Care Services/	32420
4	Physicians, Family/	16068
5	Physicians, Primary Care/	3026
6	Community Health Services/ or Community Pharmacy Services/ or Health Facilities/ or Health Facility Administration/	50698
7	Community Health Nursing/ or Home Health Nursing/ or Family Nursing/	20961
8	Community Health Workers/	4898
9	Preventive Health Services/	12985
10	Primary Health Care/	72731
11	Primary Prevention/	17642
12	Public Health/	77186
13	Rural Health Services/	12147
14	(primary care or general practi* or primary health or community mental health* or family practice or family medicine or family doctor or family physician* or home care or home based or home health* or community health* or community nurs* or health visit* or community pharmac* or preventive care or prevention program* or preventive service* or preventive health or primary prevention or public health or rural health or health promotion or health facilit*).ti,ab,kw.	570028
15	((guideline* or protocol*) adj4 (adher* or comply or complian* or observ*)).ti,ab,kw.	21273
16	((therap* or prescrib* or prescript* or diagnos*) adj2 (computer* or digital or electronic)).ti,ab,kw.	8127
17	or/1‐16	769985
18	Cell Phones/	7902
19	Smartphone/	3152
20	MP3‐Player/	178
21	Computers, Handheld/	3385
22	((cell* or mobile*) adj1 (phone* or telephone* or technolog* or device*)).ti,ab,kw.	17120
23	(handheld or hand‐held).ti,ab,kw.	11667
24	(smartphone* or smart‐phone* or cellphone* or mobiles).ti,ab,kw.	10210
25	((personal adj1 digital) or (PDA adj3 (device* or assistant*)) or MP3 player* or MP4 player*).ti,ab,kw.	1342
26	(samsung or nokia).ti,ab,kw.	1078
27	(windows adj3 (mobile* or phone*)).ti,ab,kw.	50
28	android.ti,ab,kw.	2208
29	(ipad* or i‐pad* or ipod* or i‐pod* or iphone* or i‐phone*).ti,ab,kw.	2581
30	(tablet* adj3 (device* or computer*)).ti,ab,kw.	1425
31	Telemedicine/ or Telecommunications/	24309
32	Webcasts as topic/	307
33	Text Messaging/	2335
34	Telenursing/	200
35	(mhealth or m‐health or "mobile health" or ehealth or e‐health or "electronic health" or "digital health" or uhealth or u‐health).ti,ab,kw.	24071
36	(telemedicine or tele‐medicine or telehealth or tele‐health or telecare or tele‐care or telenursing or tele‐nursing or telepsychiatry or tele‐psychiatry or telemonitor* or tele‐monitor* or teleconsult* or tele‐consult* or telecounsel* or tele‐counsel* or telecoach* or tele‐coach*).ti,ab,kw.	16741
37	(webcast* or web‐cast*).ti,ab,kw.	242
38	(((text* or short or voice or multimedia or multi‐media or electronic or instant) adj1 messag*) or instant messenger).ti,ab,kw.	4787
39	(texting or texted or texter* or ((sms or mms) adj (service* or messag*)) or interactive voice response* or IVR or voice call* or callback* or voice over internet or VOIP).ti,ab,kw.	3144
40	(Facebook or Twitter or Whatsapp* or Skyp* or YouTube or "You Tube" or Google Hangout*).ti,ab,kw.	6771
41	Mobile Applications/	4379
42	"mobile app*".ti,ab,kw.	3694
43	Reminder Systems/	3247
44	(remind* adj3 (text* or system* or messag*)).ti,ab,kw.	1673
45	Medical informatics/ or Medical informatics applications/	13493
46	Nursing informatics/ or Public health informatics/	2601
47	((medical or clinical or health or healthcare or nurs*) adj3 informatics).ti,ab,kw.	5321
48	Computer‐Assisted Instruction/	11542
49	((interactive or computer‐assisted) adj1 (tutor* or technolog* or learn* or instruct* or software or communication)).ti,ab,kw.	2423
50	or/18‐49	134198
51	sd.fs.	65061
52	exp Vaccines/ec, st, sd [Economics, Standards, Supply & Distribution]	10938
53	[exp "Equipment and Supplies"/ec, sn, sd, td, ut [Economics, Statistics & Numerical Data, Supply & Distribution, Trends, Utilization]]	0
54	(exp Therapeutic Uses/ec, st, sd or exp Pharmaceutical Preparations/ec, st, sd, ut) not Veterinary Drugs/	50819
55	pharmaceutical services/ or community pharmacy services/ or drug information services/ or pharmaceutical services, online/ or pharmacy service, hospital/	26568
56	hospital distribution systems/ or materials management, hospital/ or inventories, hospital/ or medication systems, hospital/ or product line management/	9650
57	((commodit* or consumable* or stock or stocks or supply or supplies) adj3 (inventor* or level* or notif* or track* or count* or report* or chain or out or outs or manag* or order* or logistic* or system or systems or shortage* or manag* or monitor* or maintain* or maintenance or audit or auditing)).ti,ab,kw.	12107
58	((health or medical or medicines or vaccine* or drug or drugs or laborator* or diagnos*) adj3 (product* or supply or supplies or consumable* or commodit* or stock or stocks or stockout* or "stock out*" or shortage*)).ti,ab,kw.	39432
59	health resources/ or clinical laboratory information systems/ or clinical pharmacy information systems/ or database management systems/ or hospital information systems/ or ambulatory care information systems/ or pharmacy administration/ or drug utilization/ or "drug utilization review"/ or drug storage/mt	58923
60	or/51‐59	243418
61	17 and 50 and 60	1058

**Embase Ovid (1974 to 2019 Week 31)**  

**Table:**

**#**	**Searches**	**Results**
1	mobile phone/ or smartphone/	24225
2	mp3 player/	190
3	((cell* or mobile*) adj1 (phone* or telephone* or technolog* or device*)).ti,ab,kw.	22019
4	(handheld or hand‐held).ti,ab,kw.	16056
5	(smartphone* or smart‐phone* or cellphone* or mobiles).ti,ab,kw.	14147
6	((personal adj1 digital) or (PDA adj3 (device* or assistant*)) or MP3 player* or MP4 player*).ti,ab,kw.	1835
7	(samsung or nokia).ti,ab,kw.	1974
8	(windows adj3 (mobile* or phone*)).ti,ab,kw.	75
9	android.ti,ab,kw.	3493
10	(ipad* or i‐pad* or ipod* or i‐pod* or iphone* or i‐phone*).ti,ab,kw.	4807
11	(tablet* adj3 (device* or computer*)).ti,ab,kw.	2269
12	telemedicine/ or telecardiology/ or teleconsultation/ or teledermatology/ or telediagnosis/ or telemonitoring/ or telepathology/ or telepsychiatry/ or teleradiotherapy/ or telesurgery/ or teletherapy/	33993
13	videoconferencing/ or webcast/	3709
14	text messaging/	4233
15	telenursing/	250
16	(mhealth or m‐health or "mobile health" or ehealth or e‐health or "electronic health").ti,ab,kw.	31128
17	(telemedicine or tele‐medicine or telehealth or tele‐health or telecare or tele‐care or telenursing or tele‐nursing or telepsychiatry or tele‐psychiatry or telemonitor* or tele‐monitor* or teleconsult* or tele‐consult* or telecounsel* or tele‐counsel* or telecoach* or tele‐coach*).ti,ab,kw.	22761
18	(videoconferenc* or video‐conferenc* or webcast* or web‐cast*).ti,ab,kw.	4192
19	(((text* or short or voice or multimedia or multi‐media or electronic or instant) adj1 messag*) or instant messenger).ti,ab,kw.	6359
20	(texting or texted or texter* or ((sms or mms) adj (service* or messag*)) or interactive voice response* or IVR or voice call* or callback* or voice over internet or VOIP).ti,ab,kw.	4547
21	(Facebook or Twitter or Whatsapp* or Skyp* or YouTube or "You Tube" or Google Hangout*).ti,ab,kw.	9840
22	mobile application/	8609
23	"mobile app*".ti,ab,kw.	4409
24	social media/	16159
25	(social adj (media or network*)).ti,ab,kw.	30367
26	reminder system/	2462
27	(remind* adj3 (text* or system* or messag*)).ti,ab,kw.	2445
28	e‐mail/	19439
29	(electronic mail* or email* or e‐mail or webmail).ti,ab,kw.	28880
30	medical informatics/	19671
31	nursing informatics/	1490
32	((medical or clinical or health or healthcare or nurs*) adj3 informatics).ti,ab,kw.	8448
33	multimedia/	3719
34	hypermedia/	379
35	blogging/	293
36	(multimedia or multi‐media or hypermedia or hyper‐media or blog* or vlog* or weblog* or web‐log*).ti,ab,kw.	10569
37	teaching/	86119
38	((interactive or computer‐assisted) adj1 (tutor* or technolog* or learn* or instruct* or software or communication)).ti,ab,kw.	3524
39	or/1‐38	316531
40	mental health service/	52796
41	general practice/	75463
42	home care/ or home mental health care/ or visiting nursing service/	58778
43	general practitioner/	93191
44	community health nursing/ or community psychiatric nursing/	23363
45	community care/	52617
46	pharmacy/	69572
47	health care facility/	65950
48	family nursing/	1312
49	health auxiliary/	6155
50	preventive health service/	26273
51	primary health care/	62391
52	primary prevention/	38623
53	public health/	164088
54	rural health care/ or rural health nursing/	13138
55	(primary care or general practi* or primary health or community mental health* or family practice or family medicine or family doctor or family physician* or home care or home based or home health* or community health* or community nurs* or health visit* or community pharmac* or preventive care or prevention program* or preventive service* or preventive health or primary prevention or public health or rural health or health promotion or health facilit*).ti,ab,kw.	698182
56	or/40‐55	1113544
57	vaccine/	55988
58	devices/	94990
59	drug/	37656
60	hospital pharmacy/	13501
61	device material/	845
62	inventory control/ or stock assessment/	1199
63	hospital organization/	10246
64	hospital management/	43468
65	((commodit* or consumable* or stock or stocks or inventor* or supply or supplies) adj3 (level* or notif* or track* or count* or report* or chain or out or outs or manag* or order* or logistic* or system or systems or shortage* or manag* or monitor* or maintain* or maintenance or audit or auditing)).ti,ab,kw.	21884
66	((health or medical or medicines or vaccine* or drug or drugs or laborator* or diagnos*) adj3 (product* or supply or supplies or consumable* or commodit* or stock or stocks or stockout* or "stock out*" or shortage*)).ti,ab,kw.	53622
67	health care planning/	93957
68	information system/	36410
69	materials management/ or management/ or resource management/	59769
70	drug storage/	11427
71	drug utilization/ or "drug utilization review"/	19706
72	or/57‐71	526370
73	39 and 56 and 72	4221
74	limit 73 to embase	1672

**POPLINE, K4Health (searched 07 August 2019)**

All Fields:

((commodit* OR consumable* OR stock OR stocks OR inventor* OR supply OR supplies) AND (level* OR notif* OR track* OR count* OR report* OR chain OR out OR outs OR manag* OR order* OR logistic* OR system OR systems OR shortage* OR manag* OR monitor* OR maintain* OR maintenance OR audit OR auditing))

**OR**

All Fields:

((health OR medical OR medicines OR vaccine* OR drug OR drugs OR laborator* OR diagnos*) AND (product* OR supply OR supplies OR consumable* OR commodit* OR stock OR stocks OR stockout* OR "stock out*" OR shortage*))

**OR**

Keywords:

EQUIPMENT AND SUPPLIES OR DRUGS OR VACCINES OR INVENTORIES

**AND**

All Fields:

((cell OR cellular OR mobile) AND (phone OR phones OR telephone OR telephones OR technology OR technologies OR device OR devices)) OR smartphone OR smartphones OR smart‐phone OR smart‐phones OR cellphone OR cellphones OR mobiles OR mhealth OR m‐health OR "mobile health" OR ehealth OR e‐health OR "electronic health" OR telemedicine OR tele‐medicine OR telehealth OR tele‐health OR telecare OR tele‐care OR telenursing OR tele‐nursing OR telepsychiatry OR tele‐psychiatry OR telemonitor OR telemonitoring OR tele‐monitor OR tele‐monitoring OR teleconsult OR teleconsulting OR tele‐consult OR tele‐consulting OR telecounsel OR telecounseling OR tele‐counsel OR tele‐counseling OR telecoach OR telecoaching OR tele‐coach OR tele‐coaching OR videoconference OR videoconferences OR videoconferencing OR video‐conference OR video‐conferences OR video‐conferencing OR webcast OR webcasts OR webcasting OR web‐cast OR web‐casts OR web‐casting OR ((text OR texts OR texting OR short OR voice OR multimedia OR multi‐media OR electronic OR instant) AND (message OR messages OR messaging)) OR "instant messenger" OR texting OR texted OR texter OR texters OR ((sms OR mms) AND (service OR services OR message OR messages OR messaging)) OR "interactive voice response" OR "interactive voice responses" OR ivr OR "voice call" OR "voice calls" OR callback OR "voice over internet" OR voip OR "mobile app" OR "mobile apps" OR "mobile application" OR "mobile applications" OR "social media" OR ((medical OR clinical OR health OR healthcare OR nurse OR nurses OR nursing) AND informatics)

**OR**

Keywords:

TEXT MESSAGING OR MOBILE DEVICES OR INFORMATION COMMUNICATION TECHNOLOGY OR CELLULAR PHONE

**Global Index Medicus, WHO (searched 07 August 2019)**

(tw:(("cell phone" OR "cell phones" OR "cellular phone" OR "cellular phones" OR "mobile phone" OR "mobile phones" OR "mobile devices" OR "mobile devices" OR smartphone OR smartphones OR smart‐phone OR smart‐phones OR cellphone OR cellphones) )) OR (mh:(("cell phones" OR smartphone OR mp3‐player OR "Computers, Handheld" OR telemedicine OR videoconferencing OR "Text Messaging" OR telenursing OR "Mobile Applications" OR "Reminder Systems" OR "Electronic Mail" OR "Medical Informatics" OR "Nursing Informatics" OR "Public Health Informatics" OR multimedia OR hypermedia OR blogging OR telemedicine))) AND (mh:(vaccines OR equipment OR therapeutic OR "pharmaceutical services" OR "community pharmacy services" OR "drug information services" OR "hospital distribution systems" OR "materials management, hospital" OR "inventories, hospital" OR "medication systems, hospital" OR "product line management" OR "health resources" OR "clinical laboratory information systems" OR "clinical pharmacy information systems" OR "database management systems" OR "hospital information systems" OR "ambulatory care information systems" OR "medication systems, hospital" OR "pharmacy administration" OR "drug utilization" OR "drug utilization review" OR "drug storage"))

OR

(tw:("cell phone" OR "cell phones" OR "cellular phone" OR "cellular phones" OR "mobile phone" OR "mobile phones" OR "mobile devices" OR "mobile devices" OR smartphone OR smartphones OR smart‐phone OR smart‐phones OR cellphone OR cellphones)) OR (mh:("cell phones" OR smartphone OR mp3‐player OR "Computers, Handheld" OR telemedicine OR videoconferencing OR "Text Messaging" OR telenursing OR "Mobile Applications" OR "Reminder Systems" OR "Electronic Mail" OR "Medical Informatics" OR "Nursing Informatics" OR "Public Health Informatics" OR multimedia OR hypermedia OR blogging OR telemedicine)) AND (tw:(commodit* OR consumable* OR stock OR stocks OR inventor* OR supply OR supplies OR shortage*))

**International Clinical Trials Registry Platform (ICTRP), WHO (searched 07 August 2019)**

Two separate strategies. Used advanced search, with recruitment status: All

**Strategy 1:**

Title: commodity OR stock OR supplies OR inventory OR stockouts

AND

Intervention: mobile device OR mobiles OR smartphone OR phone OR cellphone

**Strategy 2:**

Title: mobile device OR mobiles OR smartphone OR phone OR cellphone

AND

Intervention: commodity OR stock OR supplies OR inventory OR stockouts

**ClinicalTrials.gov, NIH (searched 07 August 2019)**

Other Terms: (commodity OR stocks OR supplies OR inventory OR stockouts) AND ("mobile phone" OR "mobile phones" OR "mobile devices" OR mobiles OR smartphone OR smartphones)

### Appendix 2. WEIRD tool for studies included in the secondary objective

**Table:**

**Assessment criteria** Choose one of yes, no, or unclear
**Pre‐assessment question. Is the source material based on, or does it include, empirical data (i.e. information collected through measurement or observation)?** If yes, than also include the assessment questions highlighted in grey, below.
**Pre‐assessment question. Please select the type of source material to which the assessment tool will be applied.** Choose from the following: Description of a programme, or intervention, or policy, or reform (e.g. a health, or welfare, or environmental programme, or intervention) Description of the implementation of a programme, or intervention, or policy, or reform Description of a policy process or an aspect of this process Commentary on a programme, or intervention, or policy, or reform (e.g. a health systems or development sector policy or reform) Other (please describe):
**Is there a clearly stated aim, objective, or purpose for the source material?** Apply to all source materials. Consider the following: Does the source material state its aim, objective, or purpose clearly? If the aim, objective, or purpose is not stated clearly by the authors, can it be derived from the material?
**Is there a clear description of the source of the information reported (transparency)?** Apply to all source materials. Consider: Are the sources (key informants, own experience, research study, etc.) described? Where applicable, is there a clear description of who collected the information?
**Is there a clear description of the programme, or intervention, or policy, or reform on which the source material focuses?** Apply to all source materials that describe an intervention, or programme, or policy. Consider: Are the rationale, goals, or objectives of the programme, or intervention, or policy, or reform described? Is the content of the programme, or intervention, or policy described, including all of the important facets or elements? Are the stakeholders or groups involved in delivering the programme, or intervention, or policy described, including their characteristics, background, skills or expertise, training, and responsibilities? Is the target(s) of the programme, or intervention, or policy described? Are the methods used to implement the programme, or intervention, or policy, including the mode of delivery (e.g. face‐to‐face, via the internet) and any relevant training, described? Are any materials used in the programme or intervention described? Does the source material describe clearly any infrastructure and resources required for the programme, or intervention, or policy? Does the source material describe when the programme, or intervention, or policy was started, when it finished, its intensity, and whether there were any changes to the programme, or intervention, or policy, over time? Does the source material describe any mechanisms used to ensure that the programme, or intervention, or policy, or reform was implemented as intended (e.g. supervision and support of personnel, training, implementation checks, incentives)?
**Is there a clear description of the context(s) to which the information described in the source material relates?** Apply to all source materials. Consider: Does the source material describe where the programme took place (e.g. country name(s), specific locations, urban or rural environments)? Does the source material describe clearly the context for the material, including (where relevant): The setting (country, service, community) to which the description relates; The system (e.g. health or welfare system), including the system level (e.g. frontline level); The historical, sociocultural, socioeconomic, or ethical context; The political, legal, governance, policy, or practice context (or combination), including relevant key events or policy initiatives? Does the source material clearly describe the stakeholders to which the description relates, including (where relevant): The target population(s) or group(s) for the programme, or intervention, or policy; Implementing organization(s) for the programme, or intervention, or policy; Any other partners and stakeholders? Does the source material clearly describe how the different stakeholders were involved in the programme, or intervention, or policy, or reform?
**Is the information accurate?** Apply to source materials that include little or no empirical data. Consider: Is there a clear description of whatever is the focus of the source material? Does the information presented appear to be reasonably complete? Does the source material describe any efforts to ensure that the information presented is complete and reliable?
**Is the information accurate (*empirical studies only*)?** Only source materials that include empirical data. Consider: Does the source material have clearly stated methods, including (where relevant) the type of empirical study conducted and when the programme, or intervention, or policy was evaluated? Was the basis for selected cases, or people, or clusters appropriate for the purpose of the study? Were the methods and tools for data collection appropriate for the purpose of the study? Were the data collectors appropriately trained and supported in their tasks? When were the data collected, and was the time span of the study long enough to address the core issues fairly? Was the quality of the data collected monitored and was the quality shown to be adequate? Is the method of analysis reported clearly? Is the method of analysis appropriate for the purpose of the study? Is there a clear description of the outcome(s) measured? Is the outcome measure reliable? Were these outcomes measured appropriately? Do these outcomes provide a reasonable assessment of the issue being considered? Are the linkages between the data that were reported and any inferences made transparent?
**Is the evidence representative? (with respect to population of interest, sampling frame, etc.)** Apply to all source materials. Consider: If the evidence is drawn from a sample of the population of interest, is there a clear description of how the sampling was conducted? Was the sampling approach appropriate (where applicable)? If generalisations were made to wider population(s) or setting(s), is there a rationale for doing so and a description of how this was done?
**Are any limitations of the information, the methods, or both discussed in the source material?** Apply to all source materials.
**Is evidence provided to support any findings or conclusions made?** Apply to all source materials. Consider: Are the findings or conclusions (where applicable) supported by evidence? Are the findings or conclusions reasonable, in relation to the evidence presented?
**Are relevant rights and ethics considerations described (*empirical studies only*)?** Apply only to source materials that include empirical data. Consider whether: The source material discusses relevant rights and ethics considerations; The source material indicates whether ethics approval was sought and obtained; The source material reports how consent to provide data or information was obtained.
**Are any interests declared and any potential conflicts of interest noted?** Apply to all source materials. Consider whether: The source material indicates if any of the authors are affiliated with the organisation or entity whose programme, or intervention, or policy is described; The source of funding for developing the material is reported; The source material indicates if any of the authors are affiliated with the organisation or entity that has funded the programme or policy described; Any potential conflicts of interest are described; The author indicates how any potential conflicts of interest were addressed.
**Overall assessment: please choose one of:** No or few limitations (when the answer to most questions in the tool is yes) Minor limitations (when the answer to most questions in the tool is yes or unclear) Significant or major limitations (significant or major limitations should be chosen when the answer to one or more questions in the tool is no)
**Explanation of overall assessment** Note: minor modifications were made to this tool since it was used for this study.

## Data and analyses

**Comparison 1:** Mobile stock notification and enhanced management (cStock + EM) vs standard care

Outcome or subgroup title	No. of studies	No. of participants	Statistical method	Effect size
1.1 Stockout of drugs in the last 30 days	1		Risk Ratio (M‐H, Random, 95% CI)	Subtotals only
1.1.1 Stockout of cotrimoxazole 480 mg	1	171	Risk Ratio (M‐H, Random, 95% CI)	0.96 [0.49, 1.90]
1.1.2 Stockout of artemether‐lumefantrine 1 X 6	1	171	Risk Ratio (M‐H, Random, 95% CI)	0.66 [0.35, 1.22]
1.1.3 Stockout of artemether‐lumefantrine 2 X 6	1	171	Risk Ratio (M‐H, Random, 95% CI)	0.72 [0.36, 1.44]
1.1.4 Stockout of ORS sachets	1	171	Risk Ratio (M‐H, Random, 95% CI)	1.01 [0.61, 1.69]
1.1.5 Stockout of zinc 20 mg	1	171	Risk Ratio (M‐H, Random, 95% CI)	0.99 [0.56, 1.78]
1.2 Stockout of drugs on the day of visit	1		Risk Ratio (M‐H, Random, 95% CI)	Subtotals only
1.2.1 Stockout of cotrimoxazole 480 mg	1	167	Risk Ratio (M‐H, Random, 95% CI)	0.83 [0.32, 2.11]
1.2.2 Stockout of artemether‐lumefantrine 1 X 6	1	167	Risk Ratio (M‐H, Random, 95% CI)	0.38 [0.14, 1.01]
1.2.3 Stockout of artemether‐lumefantrine 2 X 6	1	167	Risk Ratio (M‐H, Random, 95% CI)	1.21 [0.63, 2.32]
1.2.4 Stockout of ORS sachets	1	167	Risk Ratio (M‐H, Random, 95% CI)	1.64 [0.82, 3.29]
1.2.5 Stockout of zinc 20 mg	1	167	Risk Ratio (M‐H, Random, 95% CI)	1.17 [0.52, 2.60]

**Comparison 2:** Mobile stock notification and efficient product transport (cStock + EPT) vs standard care

Outcome or subgroup title	No. of studies	No. of participants	Statistical method	Effect size
2.1 Stockout of drugs in the last 30 days	1		Risk Ratio (M‐H, Random, 95% CI)	Subtotals only
2.1.1 Stockout of cotrimoxazole 480 mg	1	168	Risk Ratio (M‐H, Random, 95% CI)	1.31 [0.70, 2.44]
2.1.2 stockout of artemether‐lumefantrine 1 X 6	1	168	Risk Ratio (M‐H, Random, 95% CI)	1.21 [0.73, 1.99]
2.1.3 Stockout of artemether‐lumefantrine 2 X 6	1	168	Risk Ratio (M‐H, Random, 95% CI)	1.43 [0.81, 2.50]
2.1.4 Stockout of ORS sachets	1	168	Risk Ratio (M‐H, Random, 95% CI)	1.10 [0.67, 1.82]
2.1.5 Stockout of zinc 20 mg	1	168	Risk Ratio (M‐H, Random, 95% CI)	0.61 [0.30, 1.23]
2.2 Stockout of drugs on the day of visit	1		Risk Ratio (M‐H, Random, 95% CI)	Subtotals only
2.2.1 Stockout of cotrimoxazole 480 mg	1	164	Risk Ratio (M‐H, Random, 95% CI)	0.86 [0.34, 2.19]
2.2.2 Stockout of artemether‐lumefantrine 1 X 6	1	164	Risk Ratio (M‐H, Random, 95% CI)	0.71 [0.33, 1.55]
2.2.3 Stockout of artemether‐lumefantrine 2 X 6	1	164	Risk Ratio (M‐H, Random, 95% CI)	1.34 [0.71, 2.53]
2.2.4 Stockout of ORS sachets	1	164	Risk Ratio (M‐H, Random, 95% CI)	0.90 [0.39, 2.06]
2.2.5 Stockout of zinc 20 mg	1	164	Risk Ratio (M‐H, Random, 95% CI)	0.33 [0.09, 1.16]

## Characteristics of studies

### Characteristics of included studies [ordered by study ID]

**Table:** Asiimwe 2011

***Study characteristics***		
Methods	*Secondary objective:* case study using analysis of programmatic data	
Participants	336 trained frontline health workers **Context:** Gulu and Kabale districts in Uganda	
Interventions	SMS‐based reporting of stock‐related information to a central server. Data were available on a password‐protected dashboard to district management and surveillance teams	
Outcomes	N/A	
Notes

**Table:** Atnafu 2017

***Study characteristics***		
Methods	*Secondary objective*: Community‐based cluster‐randomised controlled trial with pre‐ and post‐intervention surveys	
Participants	Secondary objective 3240 mothers were surveyed across three clusters (1080 from each cluster), Health Extension Workers (HEWs), and voluntary health workers (vCHW) **Included survey participants:** for the baseline survey, women of 15 to 49 years, who had a child younger than 5 years; for the post‐intervention survey, women of 15 to 49 years, who had at least one child younger than one year. **Setting:** three woredas (districts) in Ethiopia's Guraghe zone with sufficient mobile phone network coverage (Abeshge, Ezha, Sodo)	
Interventions	1. **Partial intervention:** 48 health extension workers (HEW) received a mobile phone with locally developed, customised SMS‐based mobile phone application for filling maternal‐, child‐, and stock‐related forms linked to the central server, which in turn, sent a reminder about the scheduled date of the ANC visit, expected date of delivery, PNC, immunisation schedule, and vaccine and contraceptive stock status 2. **Full intervention:** 49 HEWs received a mobile phone with locally developed, customised SMS‐based mobile phone application for filling maternal‐, child‐, and stock‐related forms linked to the central server, which in turn, sent a reminder about the scheduled date of the ANC visit, expected date of delivery, PNC, immunisation schedule, and vaccine and contraceptive stock status + 56 volunteer community health workers (CHW) received mobile phones without the application	
Outcomes	A structured and pre‐tested questionnaire was used to collect data pre‐ (2012) and post‐ (2013) intervention. Outcomes included: response rates; ANC, delivery, and PNC service utilisation; contraceptive stockouts and utilisation rates; immunisation coverage	
Notes	**Source of funding:** World Bank and African Development Bank in collaboration with Addis Ababa university

**Table:** Barrington 2010

***Study characteristics***		
Methods	*Secondary objective:* case study using analysis of programmatic data	
Participants	Health facility health workers **Context:** three districts of rural Tanzania, involving 129 health facilities	
Interventions	Facility‐based health workers used mobile phones to send information on stock counts of four dosage packs of artemether‐lumefantrine (AL) and quinine using SMS messages, to district management team on a weekly basis. These data were made available on a secure website for the use of the district management team.	
Outcomes	N/A	
Notes	The program described here is the same intervention as the one described by Githinji 2013; Mikkelsen‐Lopez 2014; WHO 2013, with differences in the products about which stock data were reported.

**Table:** Barron 2016

***Study characteristics***		
Methods	*Secondary objective:* case study using analysis of programmatic data	
Participants	Pregnant women **Context:** South Africa	
Interventions	MomConnect provides targeted pregnancy‐related information to pregnant women via SMS. Once the women visit the health facility, they provide feedback via SMS about availability and quality of services received (including stock‐availability at the facility)	
Outcomes	N/A	
Notes

**Table:** Biemba 2017

***Study characteristics***		
Methods	*Secondary objective:* case study describing program implementation	
Participants	40 Community Health Workers (CHW) and 20 CHW supervisors **Context:** Chipata and Chadiza districts in Zambia	
Interventions	CHWs used phones to report on integrated Community Case Management (iCCM) cases seen, managed, and referred, and iCCM medical and diagnostic supplies received and dispensed.	
Outcomes	N/A	
Notes	Information used was derived from authors’ implementation experience

**Table:** Chandani 2017

***Study characteristics***		
Methods	*Secondary objective:* case study describing program implementation and selective results from routine evaluations	
Participants	Community health workers (CHWs), health centre staff, and district managers **Context:** Malawi and Rwanda	
Interventions	Stock reporting by CHWs using ‘cStock’, plus quality improvement (QI) approaches to ensure that health centre and district‐level staff reviewed and responded to stock data	
Outcomes	N/A	
Notes	Study design for the evaluations not described. The intervention referred to is the same as the one described in Shieshia 2014, in Malawi

**Table:** Githinji 2013

***Study characteristics***		
Methods	*Secondary objective:* case study using programmatic data	
Participants	Facility‐based health workers **Context:** 87 public health facilities in 5 Kenyan districts	
Interventions	Health workers sent information on stock counts of artemether‐lumefantrine (AL) and rapid diagnostic tests (RDT) using SMS messages through their mobile phones to a web‐based system accessed by district managers.	
Outcomes	N/A	
Notes	The program described here, SMS for Life, is the same intervention as the one described by Barrington 2010; Mikkelsen‐Lopez 2014; WHO 2013, with differences in the products about which stock data were reported.

**Table:** Hara 2017

***Study characteristics***		
Methods	*Secondary objective:* case study describing program implementation	
Participants	Not reported **Context:** Ethiopia and Pakistan	
Interventions	In Pakistan, the use of global standards‐based bar codes for inventory management of contraceptive supplies. In Ethiopia, a smart phone application was used to scan the bar codes using the mobile phone camera. These data were collated in a central inventory management system.	
Outcomes	N/A	
Notes

**Table:** Mikkelsen‐Lopez 2014

***Study characteristics***		
Methods	*Secondary objective:* case study using programmatic data	
Participants	Health facility workers **Context:** 5000 public health facilities in Tanzania	
Interventions	Facility‐based health workers used mobile phones to send information on stock counts of four dosage packs of artemether‐lumefantrine (AL), using SMS messages within 27 hours of receiving a reminder, on a weekly basis. These data were made available as summary reports to the District Medical Officer and District Pharmacist.	
Outcomes	N/A	
Notes	The program described here, SMS for Life, is the same intervention as the one described by Barrington 2010; Githinji 2013; WHO 2013, with differences in the products about which stock data were reported.

**Table:** Namisango 2016

***Study characteristics***		
Methods	*Secondary objective:* case study using programmatic data	
Participants	Clinic staff (health professional and pharmacist), palliative care patients **Context:** rural hospital and urban hospice in Uganda	
Interventions	A software application that links patient records with pharmacy records to facilitate routine reporting and dispensing of medicine.	
Outcomes	N/A	
Notes

**Table:** Negandhi 2016

***Study characteristics***		
Methods	*Secondary objective:* case study using programmatic data	
Participants	District immunization officer, medical faculty and students, cold chain technician **Context:** Bihar, India	
Interventions	Real‐time capture of vaccine stocks by health facility staff, followed by submission to a central server, and data availability on a dashboard.	
Outcomes	N/A	
Notes	We found the description of the intervention was unclear

**Table:** Shieshia 2014

***Study characteristics***		
Methods	*Primary objective*: the study used a controlled before‐after design, with baseline and follow‐up assessments in May 2010 and February 2013. Data collection was carried out in six districts. *Secondary objective*: the study used a combination of staff interviews, and presented a case study based on program implementation	
Participants	Health Surveillance Assistants (HSA), tasked with community case management of sick children, in six districts in Malawi. Of 393 HSAs assigned to one intervention (cStock + enhanced management), 56 were sampled at baseline and 81 at follow‐up. Of the 253 HSAs assigned to the second intervention (cStock + efficient product transport), 44 were sampled at baseline, and 78 at follow‐up. Setting: Health facilities in 10 districts in Malawi	
Interventions	**1. Intervention group A (cStock + enhanced management (EM))** cStock, a mHealth tool for community–level reporting of stock on hand data, and resupply of 19 health products managed by Health Surveillance Assistants (HSAs). cStock is an SMS and web–based reporting and resupply system that is used by HSAs to report stock data, via SMS through their personal mobile phones. cStock calculates HSA resupply quantities, and sends this information to Health Facility (HF) staff to use to pick and pack products for HSAs, and notify them of a collection time. The EM intervention addressed challenges related to data availability and visibility, and low motivation among HSAs. The additional component of the EM intervention was District Product Availability Teams (DPATs). These are multilevel quality improvement teams that use the data supplied by cStock to monitor performance of the supply chain, and make informed supply chain decisions. **2. Intervention group B (cStock + efficient product transport (EPT))** cStock, a mHealth tool for community–level reporting of stock on hand data, and resupply of 19 health products managed by Health Surveillance Assistants (HSAs). cStock is an SMS and web–based reporting and resupply system that is used by HSAs to report stock data, via SMS through their personal mobile phones. cStock calculates HSA resupply quantities, and sends this information to Health Facility (HF) staff to use to pick and pack products for HSAs, and notify them of a collection time. The EPT intervention addressed challenges of transport, plus data visibility. The additional components of the EPT intervention was training all HSAs on bicycle maintenance, providing a basic tool kit, and using a continuous review inventory control system. **3. Control group:** No intervention	
Outcomes	**Feasibility:** the feasibility of cStock was evaluated by looking at staff capacity to use cStock, practicability, and relevance **Acceptability:** the acceptability of cStock was evaluated by looking at its level of routine use, its effect on users’ daily work, and perceived benefits identified by the user. **Effectiveness:** findings on effectiveness were presented as a comparison of supply chain performance between the EM + cStock and EPT + cStock groups, according to these four indicators: reporting, complete reporting, lead time, and stockout rates over time, using data from cStock dashboard reports.	
Notes	No feasibility or acceptability data were available for the control groups. No effectiveness data were available that compared cStock to no intervention. **Source of funding**: Bill and Melinda Gates Foundation

**Table:** Stanton 2016

***Study characteristics***		
Methods	*Secondary objective:* case study describing program implementation	
Participants	Facility‐based health workers, Health Surveillance Assistants (HSA) **Context:** Tanzania, Malawi, Ghana, Ethiopia, Bangladesh	
Interventions	Liverpool mHealth Suite (LMS) used SMS service and mobile apps for neglected tropical diseases, such as lymphatic filariasis: MeasureSMS‐MDA was used to support real‐time data collection on mass drug administration coverage at village‐ and health centre‐level. MeasureSMS‐Morbidity was used to report clinical case numbers of those who received specific treatment. The LyMSS app allowed health workers to submit regular inventory reports. Data were collated in a central server, and made available on a web‐browser.	
Outcomes	N/A	
Notes

**Table:** USAID 2010

***Study characteristics***		
Methods	*Secondary objective:* case study describing program implementation	
Participants	Data collectors **Context:** Tanzania, Ghana, Liberia, Zambia	
Interventions	Data collectors used EpiSurveyor to gather stock availability and case management data for malaria medicines at health facilities each quarter.	
Outcomes	N/A	
Notes	USAID DELIVER Project Report

**Table:** USAID 2016

***Study characteristics***		
Methods	*Secondary objective:* case study describing program implementation	
Participants	Health facility staff **Context:** 24 districts, 259 health facilities, and district health offices in Zambia	
Interventions	Mobile phones used to collect and report data on stocks, storage conditions, and order fulfilment at health facilities	
Outcomes	N/A	
Notes	Meeting presentation, additional documentation identified. Under USAID DELIVER Project

**Table:** WHO 2013

***Study characteristics***		
Methods	*Secondary objective:* case study describing program implementation	
Participants	Health facility workers **Context:** Tanzania, Cameroon, Ghana, Kenya, Chad, Democratic Republic of Congo	
Interventions	SMS for Life sent weekly SMS to health facilities requesting stock information. Facility‐based health workers used mobile phones to send information on relevant stocks using SMS messages, on a weekly basis. These data were made available as summary reports to the District Medical Officer and District Pharmacist, who could make decision about transfer of commodities.	
Outcomes	N/A	
Notes	The program described here is the same intervention as the one described by Barrington 2010; Githinji 2013; Mikkelsen‐Lopez 2014, with differences in the products about which stock data were reported.	

### Characteristics of excluded studies [ordered by study ID]

**Table:**

Study	Reason for exclusion
Calabrese 2012	Irrelevant intervention: did not involve notification or tracking of stock levels.Tracking of medication location from order to delivery. Also, unclear if mobile devices were used ('web‐based') and study was not set in primary care.
Chaffee 2010	Irrelevant study design: editorial, narrative review on computerised prescriber order entry system alerts
Chiu 2019	Irrelevant intervention: did not involve notification or tracking of stock levels
Fisher 2018	Irrelevant intervention: not accessible by, nor primarily used by mobile
Hazel 2015	Irrelevant study design: literature review of malaria research in Malawi
Nzolo 2018	Irrelevant intervention: did not involve notification or tracking of stock levels
Okoli 2015	Irrelevant study design: Measured outcomes once before and 3 times after intervention, study was not controlled
Patel 2006	Irrelevant study design: described pilot and production phases of development of pharmacy tool, no control group or comparison was made
Peek 2016	Irrelevant study design: not primary care
Rao 2013	Irrelevant study design: narrative review
Tamblyn 2010	Irrelevant intervention: did not involve notification or tracking of stock levels
Umlauf 2017	Irrelevant study design: paper reports on handling of stockout situations, no evaluation of any intervention
Williams 2018	Irrelevant intervention: did not involve notification or tracking of stock levels
Wolfe 2017	Irrelevant study design: commentary, advice, guideline on pharmacy data management
Zablotska 2018	Irrelevant study design: editorial

## Differences between protocol and review

In the protocol, we had proposed using the SURE framework to synthesise secondary findings. However, we decided not to use this framework in the review as it did not fit the data well.

In the protocol, we did not indicate how we would attempt to integrate the findings from the primary and secondary review objectives. This was added during the review process.

We use the Ways of Evaluating Important and Relevant Data (WEIRD) tool to assess the methodological limitations of the studies included for the secondary review objective (Lewin 2019). This is a new tool, which was not available when the protocol was developed.

## References

[CD012907-bib-0001] Asiimwe C, Gelvin D, Lee E, Ben Amor Y, Quinto E, Katureebe C, et al. Use of an innovative, affordable, and open-source short message service-based tool to monitor malaria in remote areas of Uganda. American Journal of Tropical Medicine and Hygiene 2011;85(1):26-33. 10.4269/ajtmh.2011.10-0528 PMC3122339 21734120

[CD012907-bib-0002] Atnafu A, Otto K, Herbst CH. The role of mHealth intervention on maternal and child health service delivery: findings from a randomized controlled field trial in rural Ethiopia. mHealth 2017 Sep 14;3:39. [DOI: 10.21037/mhealth.2017.08.04] PMC5682387 29184891

[CD012907-bib-0003] Barrington J, Wereko-Brobby O, Ward P, Mwafongo W, Kungulwe S. SMS for Life: a pilot project to improve anti-malarial drug supply management in rural Tanzania using standard technology. Malaria Journal 2010;9:298. 10.1186/1475-2875-9-298 PMC2978233 20979633

[CD012907-bib-0004] Barron P, Pillay Y, Fernandes A, Sebidi J, Allen R. The MomConnect mHealth initiative in South Africa: early impact on the supply side of MCH services. Journal of Public Health Policy 2016;37(2):S201-12. 10.1057/s41271-016-0015-2 27899795

[CD012907-bib-0005] Biemba G, Chiluba B, Yeboah-Antwi K, Silavwe V, Lunze K, Mwale RK, et al. A mobile-based community health management information system for community health workers and their supervisors in 2 districts of Zambia. Gloabl Health Science and Practice 2017;5(3):486-94. 10.9745/GHSP-D-16-00275 PMC5620344 28855233

[CD012907-bib-0006] Chandani Y, Duffy M, Lamphere B, Noel M, Heaton A, Andersson S. Quality improvement practices to institutionalize supply chain best practices for iCCM: evidence from Rwanda and Malawi. Research in Social and Administrative Pharmacy 2017;13:1095-109. 10.1016/j.sapharm.2016.07.003 PMC5704638 27567145

[CD012907-bib-0007] Githinji S, Kigen S, Memusi D, Nyandigisi A, Mbithi AM, Wamari A, et al. Reducing stock-outs of life saving malaria commodities using mobile phone text-messaging: *SMS for Life s*tudy in Kenya. PloS One 2013;8(1):e54066. 10.1371/journal.pone.0054066 PMC3547931 23349786

[CD012907-bib-0008] Hara L, Guirguis R, Hummel K, Villanueva M. More than bar codes: integrating global standards-based bar code technology into national health information systems in Ethiopia and Pakistan to increase end-to-end supply chain visibility. Global Health Science and Practice 2017;5(4):678-85. 10.9745/GHSP-D-16-00350 PMC5752613 29284701

[CD012907-bib-0009] Mikkelsen-Lopez I, Shango W, Barrington J, Ziegler R, Smith T, deSavigny D. The challenge to avoid anti-malarial medicine stock-outs in an era of funding partners: the case of Tanzania. Malaria Journal 2014;13:181. 10.1186/1475-2875-13-181 PMC4030285 24885420

[CD012907-bib-0010] Namisango E, Ntege C, Luyirika EBK, Kiyange F, Allsop MJ. Strengthening pharmaceutical systems for palliative care services in resource limited settings: piloting a mHealth application across a rural and urban setting in Uganda. BMC Palliative Care 2016;15(20):1-11. 10.1186/s12904-016-0092-9 PMC4759774 26895882

[CD012907-bib-0011] Negandhi P, Chauhan M, Mukherjee Das A, Neogi SB, Sharma J, Sethy G. Mobile-based effective vaccine management tool: an m-health initiative implemented by UNICEF in Bihar. Indian Journal of Public Health 2016;60(4):334-40. 10.4103/0019-557X.195869 27976659

[CD012907-bib-0012] Shieshia M, Noel M, Andersson S, Felling B, Alva S, Agarwal S, et al. Strengthening community health supply chain performance through an integrated approach: using mHealth technology and multilevel teams in Malawi. Journal of Global Health December 2014;4(2):020406. [DOI: 10.7189/jogh.04.020406] PMC4267094 25520796

[CD012907-bib-0013] Stanton M, Molineux A, Mackenzie C, Kelly-Hope L. Mobile technology for empowering health workers in underserved communities: new approaches to facilitate the elimination of neglected tropical diseases. JMIR Public Health and Surveillance 2016;2(1):1-11. 10.2196/publichealth.5064 PMC4869228 27227155

[CD012907-bib-0014] US Agency for International Development (USAID). Innovative mobile phone use improves access to drugs and medical supplies in Africa. USAID Deliver Project October 2010.

[CD012907-bib-0015] US Agency for International Development (USAID). USAID Deliver Project final country report: Zambia. Task Orders 4 and 7. USAID Deliver Project 2016.

[CD012907-bib-0016] World Health Organization (WHO). Preventing stock-outs of antimalarial drugs in sub-Saharan Africa: Novartis's SMS for Life. Available at apps.who.int/iris/handle/10665/92817 2013.

[CD012907-bib-0017] Calabrese SV, Williams JP. Implementation of a web-based medication tracking system in a large academic medical center. American Journal of Health-system Pharmacy 2012;69(19):1651-8. 10.2146/ajhp110527 22997118

[CD012907-bib-0018] Chaffee BW. Future of clinical decision support in computerized prescriber order entry. American Journal of Health-system Pharmacy 2010;67(11):932-5. 10.2146/ajhp090194 20484218

[CD012907-bib-0019] Chiu E, Ho C, Cheng R. Medication safety expertise at your fingertips: Medication Incident Analysis Knowledge Mobilization Tool. Canadian Pharmacists Journal 2019;152(4):223-7. 10.1177/1715163519852972 PMC6610503 31320955

[CD012907-bib-0020] Fisher AM, Mtonga TM, Espino JU, Jonkman LJ, Connor SE, Cappella NK, et al. User-centered design and usability testing of RxMAGIC: a prescription management and general inventory control system for free clinic dispensaries. BMC Health Services Research 2018;18(1):703. 10.1186/s12913-018-3517-8 PMC6131751 30200939

[CD012907-bib-0021] Hazel E, Amouzou A, Park L, Banda B, Chimuna T, Guenther T, et al. Real-time assessments of the strength of program implementation for community case management of childhood illness: validation of a mobile phone-based method in Malawi. American Journal of Tropical Medicine and Hygiene 2015;92(3):660-5. 10.4269/ajtmh.14-0396 PMC4350569 25582691

[CD012907-bib-0022] Nzolo D, Engo Biongo A, Kuemmerle A, Lusakibanza M, Lula Y, Nsengi N, et al. Safety profile of fractional dosing of the 17DD yellow fever vaccine among males and females: experience of a community-based pharmacovigilance in Kinshasa, DR Congo. Vaccine 2018;36(41):6170-82. 10.1016/j.vaccine.2018.08.052 30190119

[CD012907-bib-0023] Okoli U, Oduenyi C, Onwudinjo N, Ejeckam C, Adegoke F, Holmlund M, et al. Engaging communities in commodity stock monitoring using telecommunication technology in primary health care facilities in rural Nigeria. Health Services Research and Managerial Epidemiology 2015;2:2333392815609143. 10.1177/2333392815609143 PMC5266446 28462267

[CD012907-bib-0024] Patel RJ, Lyman AE Jr, Clark DR, Hartman TJ, Chester EA, Kicklighter CE. Personal digital assistants for documenting primary care clinical pharmacy services in a health maintenance organization. American Journal of Health-system Pharmacy 2006;63(3):258-61. 10.2146/ajhp050191 16434785

[CD012907-bib-0025] Peek G, Campbell U, Kelm M. Impact of medication dose tracking technology on nursing practice. Hospital Pharmacy 2016;51(8):646-53. 10.1310/hpj5108-646 PMC5030876 27698504

[CD012907-bib-0026] Rao VB, Schellenberg D, Ghani AC. Overcoming health systems barriers to successful malaria treatment. Trends in Parasitology 2013;29(4):164-80. 10.1016/j.pt.2013.01.005 23415933

[CD012907-bib-0027] Tamblyn R, Reidel K, Huang A, Taylor L, Winslade N, Bartlett G, et al. Increasing the detection and response to adherence problems with cardiovascular medication in primary care through computerized drug management systems: a randomized controlled trial. Medical Decision Making 2010;30(2):176-88. 10.1177/0272989X09342752 19675319

[CD012907-bib-0028] Umlauf R, Park SJ. Stock-outs! Improvisations and processes of infrastructuring in Uganda's HIV/Aids and malaria programmes. Global Public Health 2017;13(3):1-14. 10.1080/17441692.2017.1414287 29243574

[CD012907-bib-0029] Williams R, Keers R, Gude WT, Jeffries M, Davies C, Brown B, et al. SMASH! The Salford medication safety dashboard. Journal of Innovation in Health Informatics 2018;25(3):183-93. 10.14236/jhi.v25i3.1015 30398462

[CD012907-bib-0030] Wolfe A, Hess L, La MK, Pappas AL, Moore R, Granko R, et al. Strategy for pharmacy data management. American Journal of Health-system Pharmacy 2017;74(2):79-85. 10.2146/ajhp150694 28069682

[CD012907-bib-0031] Zablotska IB, Baeten JM, Phanuphak N, McCormack S, Ong J. Getting pre-exposure prophylaxis (PrEP) to the people: opportunities, challenges and examples of successful health service models of PrEP implementation. Sexual Health 2018;15(6):481-4. 10.1071/SH18182 30496716

[CD012907-bib-0032] Agarwal S, LeFevre AE, Lee J, L’Engle K, Mehl G, Sinha C, et al. Guidelines for reporting of health interventions using mobile phones: mobile health (mHealth) evidence reporting and assessment (mERA) checklist. BMJ 2016;352:i1174. 10.1136/bmj.i1174 26988021

[CD012907-bib-0033] Aranda-Jan CB, Mohutsiwa-Dibe N, Loukanova S. Systematic review on what works, what does not work and why of implementation of mobile health (mHealth) projects in Africa. BMC Public Health 2014;14:188. 10.1186/1471-2458-14-188 PMC3942265 24555733

[CD012907-bib-0034] Awofeso N. What is the difference between ’primary care’ and ’primary healthcare’? Quality in Primary Care 2004;12:93-4.

[CD012907-bib-0035] Chuma J, Okungu V, Molyneux C. Barriers to prompt and effective malaria treatment among the poorest population in Kenya. Malaria Journal 2010;9:144. 10.1186/1475-2875-9-144 PMC2892503 20507555

[CD012907-bib-0036] Damschroder L, Hall C, Gillon L, Caitlin R, Sparks J, Lowery J. The Consolidated Framework for Implementation Research (CFIR): progress to date, tools and resources, and plans for the future. Implementation Science Dec 2015;10(1):1.

[CD012907-bib-0037] Cochrane Effective Practice and Organisation of Care (EPOC). Data collection form. EPOC resources for review authors, 2017. Available from epoc.cochrane.org/epoc-specific-resources-review-authors (accessed 14 December 2017).

[CD012907-bib-0038] Cochrane Effective Practice and Organisation of Care (EPOC). Suggested risk of bias criteria for EPOC reviews. EPOC resources for review authors, 2017. Available from epoc.cochrane.org/epoc-specific-resources-review-authors (accessed 14 December 2017).

[CD012907-bib-0039] Cochrane Effective Practice and Organisation of Care (EPOC). Analysis in EPOC reviews. EPOC resources for review authors, 2017. Available from epoc.cochrane.org/resources/epoc-resources-review-authors (accessed 14 December 2017).

[CD012907-bib-0040] Cochrane Effective Practice and Organisation of Care (EPOC). EPOC worksheets for preparing a 'Summary of findings' table using GRADE. EPOC resources for review authors, 2017. Available from epoc.cochrane.org/epoc-specific-resources-review-authors (accessed 14 December 2017).

[CD012907-bib-0041] Cochrane Effective Practice and Organisation of Care (EPOC). Reporting the effects of an intervention in EPOC reviews. EPOC resources for review authors, 2017. Available from epoc.cochrane.org/resources/epoc-resources-review-authors (accessed 14 December 2017).

[CD012907-bib-0042] Frøen JF, Myhre SL, Frost MJ, Chou D, Mehl G, Say L, et al. eRegistries: electronic registries for maternal and child health. BMC Pregnancy and Childbirth 2016;16:11. 10.1186/s12884-016-0801-7 PMC4721069 26791790

[CD012907-bib-0043] Glenton C, Sorhaindo AM, Ganatra B, Lewin S. Implementation considerations when expanding health worker roles to include safe abortion care: a five-country case study synthesis. BMC Public Health 2017;17(1):730. 10.1186/s12889-017-4764-z PMC5609023 28934942

[CD012907-bib-0044] Global Health Watch. Primary health care: a review and critical appraisal of its revitalization. Available from www.ghwatch.org/sites/www.ghwatch.org/files/B1_0.pdf (accessed 1 October 2017).

[CD012907-bib-0045] GRADEpro GDT. Version accessed 15 October 2017. Hamilton (ON): McMaster University (developed by Evidence Prime), 2015. Available at gradepro.org.

[CD012907-bib-0046] Guyatt GH, Oxman AD, Vist G, Kunz R, Falck-Ytter Y, Alonso-Coello P, et al, GRADE Working Group. GRADE: an emerging consensus on rating quality of evidence and strength of recommendations. BMJ 2008;336(7650):924-6. 10.1136/bmj.39489.470347.AD PMC2335261 18436948

[CD012907-bib-0047] Higgins JP, Green S, editor(s). Cochrane Handbook for Systematic Reviews of Interventions Version 5.1.0 (updated March 2011). The Cochrane Collaboration, 2011. Available from training.cochrane.org/cochrane-handbook-systematic-reviews-interventions.

[CD012907-bib-0048] Hill J, Kazembe P. Reaching the Abuja target for intermittent preventive treatment of malaria in pregnancy in African women: a review of progress and operational challenges. Tropical Medicine & International Health 2006;11(4):409-18. 10.1111/j.1365-3156.2006.01585.x 16553924

[CD012907-bib-0049] International Telecommunications Union. ICT Facts & Figures. Available from www.itu.int/en/ITU-D/Statistics/Documents/facts/ICTFactsFigures2015.pdf (accessed 1 October 2017).

[CD012907-bib-0050] Kangwana BB, Njogu J, Wasunna B, Kedenge SV, Memusi DN, Goodman CA, et al. Malaria drug shortages in Kenya: a major failure to provide access to effective treatment. American Journal of Tropical Medicine and Hygiene 2009;80(5):737-8. PMC2679204 19407116

[CD012907-bib-0051] Lewin S, Booth A, Glenton C, Munthe-Kaas H, Rashidian A, Wainwright M, et al. Applying GRADE-CERQual to qualitative evidence synthesis findings: introduction to the series. Implementation Science 2018;2:Article number: 2. [DOI: 10.1186/s13012-017-0688-3] PMC5791040 29384079

[CD012907-bib-0052] Lewin S, Langlois, E, Tuncalp Ö, Portela A, the COMMVAC Project Team. WEIRD (Ways of Evaluating Important and Relevant Data) tool: questions to guide assessment / critical appraisal of programme descriptions, implementation descriptions and other mainly descriptive types of evidence. Available from epoc.cochrane.org/resources/epoc-resources-review-authors (accessed 28 March 2020) 2019.

[CD012907-bib-0053] Liberati A, Altman DG, Tetzlaff J, Mulrow C, Gøtzsche PC, Ioannidis JP, et al. The PRISMA statement for reporting systematic reviews and meta-analyses of studies that evaluate health care interventions: explanation and elaboration. PLoS Medicine 2009;6(7):e1000100. 10.1371/journal.pmed.1000100 PMC2707010 19621070

[CD012907-bib-0054] Mehl G (Department of Reproductive Health and Research, WHO, Geneva). [personal communication]. Conversation with: Tamrat T (Department of Reproductive Health and Research, WHO, Geneva) 5 December 2009.

[CD012907-bib-0055] Muldoon LK, Hogg WE, Levitt M. Primary care (PC) and primary health care (PHC): what is the difference? Canadian Journal of Public Health 2006;97(5):409-11. 10.1007/BF03405354 PMC6976192 17120883

[CD012907-bib-0056] Odendall WA, Watkins JA, Leon N, Goudge J, Griffiths F, Tomlinson M, et al. Health workers’ perceptions and experiences of using mHealth technologies to deliver primary healthcare services: a qualitative evidence synthesis. Cochrane Database of Systematic Reviews 2020, Issue 3. Art. No: CD011942. [DOI: 10.1002/14651858.CD011942.pub2] PMC7098082 32216074

[CD012907-bib-0057] Pasquet A, Messou E, Gabillard D, Minga A, Depoulosky A, Deuffic-Burban S, et al. Impact of drug stock-outs on death and retention to care among HIV-infected patients on combination antiretroviral therapy in Abidjan, Côte d'Ivoire. PLoS One 2010;5(10):e13414. 10.1371/journal.pone.0013414 PMC2955519 20976211

[CD012907-bib-0058] Sued O, Schreiber C, Girón N, Ghidinelli M. HIV drug and supply stock-outs in Latin America. Lancet Infectious Diseases 2011;11(11):810-1. 10.1016/S1473-3099(11)70301-2 22035613

[CD012907-bib-0059] SURE Collaboration. SURE (Supporting the Use of Research Evidence) Guides for Preparing and Using Evidence-Based Policy Briefs: 5. Identifying and addressing barriers to implementing policy options. Version 2.1. Available at www.who.int/evidence/sure/guides/en/ (accessed 7 May 2020).

[CD012907-bib-0060] Tran DN, Bero LA. Barriers and facilitators to the quality use of essential medicines for maternal health in low-resource countries: an Ishikawa framework. Journal of Global Health June 5 2015;5(1):010406. 10.7189/jogh.05.010406 PMC4416332 25969730

[CD012907-bib-0061] World Health Organization (WHO). The world medicines situation 2004. Available at apps.who.int/medicinedocs/en/d/Js6160e/9.html (accessed 10 October 2017).

[CD012907-bib-0062] World Health Organization (WHO). Primary health care: now more than ever. Available at www.who.int/whr/2008/en/ (accessed 10 October 2017).

[CD012907-bib-0063] World Health Organization (WHO). Global strategy for women’s and children’s health. Available at www.who.int/pmnch/knowledge/publications/fulldocument_globalstrategy/en/ (accessed 3 October 2017).

[CD012907-bib-0064] World Health Organization (WHO). The world medicines situation 2011 – access to essential medicines as part of the right to health. Available from apps.who.int/medicinedocs/en/d/Js18772en/ (accessed 1 October 2017).

[CD012907-bib-0065] World Health Organization (WHO). Technical consultation on preventing and managing global stock outs of medicines. Available at www.who.int/medicines/areas/access/Medicines_Shortages.pdf?ua=1 (accessed 13 August 2020).

[CD012907-bib-0066] World Health Organization (WHO). Medicine shortages: global approaches to addressing shortages of essential medicines in health systems. WHO Drug Information 2016;30(2):180-5. [apps.who.int/iris/handle/10665/331028]

[CD012907-bib-0067] World Health Organization (WHO). Meeting report: technical definitions of shortages and stockouts of medicines and vaccines. Available at www.who.int/medicines/areas/access/WHO_EMP_IAU_2017-03/en/2017.

[CD012907-bib-0068] World Health Organization. WHO Guideline: recommendations on digital interventions for health system strengthening. Available at www.who.int/reproductivehealth/publications/digital-interventions-health-system-strengthening/en/2019. 31162915

